# Drought alters jasmonate-dependent regulation of anther development in yellow lupin (*Lupinus luteus* L.)

**DOI:** 10.1007/s00425-026-05107-7

**Published:** 2026-08-01

**Authors:** Katarzyna Marciniak, Krzysztof Przedniczek, Jacek Kęsy, Andrzej Tretyn

**Affiliations:** 1 Department of Plant Physiology and Biotechnology, Faculty of Biological and Veterinary Sciences, Nicolaus Copernicus University, Lwowska 1 St, 87-100 Toruń, Poland; 2 Department of Systems Biology, Faculty of Biological and Veterinary Sciences, Nicolaus Copernicus University, Lwowska 1 St, 87-100, Toruń, Poland

**Keywords:** Anther development, Drought stress, Jasmonates, Legumes, Yellow lupin (*Lupinus luteus* L.)

## Abstract

**Main conclusion:**

Drought reshapes phytohormone dynamics and gene expression linked to jasmonate-dependent regulation of anther development, leading to delayed anther dehiscence in yellow lupin (*Lupinus luteus* L.).

**Abstract:**

Drought is a major factor limiting plant sexual reproduction and yield. Post-meiotic, late anther development involves a multistep dehiscence process leading to the opening of pollen chambers, and subsequent to degeneration of anther tissues (senescence). Effective coordination of these developmental phases is crucial for male fertility and depends on phytohormones, including jasmonates (JAs). Our morphological and histological analyses revealed that anthers of yellow lupin (*Lupinus luteus* L.) exposed to drought stress display a delayed dehiscence phenotype. The concentrations of jasmonic acid (JA) during individual developmental phases of stressed anthers were disrupted: JA levels were lower under drought during phases in which they were normally elevated under well-watered conditions, whereas they were higher during phases in which they typically declined. This imbalance correlated with JA localization in different cell types and was reflected in the expression of genes associated with phytohormone biosynthesis and perception. Transcriptomic analyses of JA-dependent interactions during specific phases of anther development identified differentially expressed genes involved in the metabolism and signaling of various phytohormones, including gibberellins (GAs), salicylic acid (SA), and indole-3-acetic acid (IAA). Moreover, drought stress altered the levels of selected phytohormones during anther dehiscence and senescence. All these findings improve our understanding of the hormonal and molecular regulatory mechanisms underlying late anther development under water deficit, contributing to breeding strategies for drought-resistant crops and the maintenance of high, stable yields.

**Supplementary Information:**

The online version contains supplementary material available at 10.1007/s00425-026-05107-7.

## Introduction

Drought is one of the most severe abiotic stresses that considerably limits plant growth, development, and productivity. Ongoing climate change is projected to modify precipitation patterns and temperature regimes, potentially increasing the duration, frequency, and intensity of water deficits in many regions, although these changes are expected to vary geographically. Plant responses to drought are often species-specific and involve diverse physiological, molecular, and developmental mechanisms (Pamungkas et al. 2022). Drought escape refers to the ability of plants to shorten the vegetative phase and accelerate flowering and seed production; drought avoidance enables plants to reduce water loss and enhance water uptake; whereas drought tolerance involves adaptive responses to water limitation at multiple levels of biological organization. Consequently, breeding for improved drought tolerance and reproductive resilience under water-limited conditions remains highly challenging. Identifying specific genes or regulatory pathways that underlie this complex trait is not straightforward. In practice, breeders face substantial difficulties when integrating multiple genetic, physiological, and biotechnological approaches to achieve meaningful improvements in drought resistance. Although extensive research has been conducted to elucidate the mechanisms underlying drought responses, much of the mechanistic insight derives from studies in the model species *Arabidopsis thaliana* (Nakashima et al. 2009), with subsequent translation to economically important crops including wheat (*Triticum aestivum* L.), rice (*Oryza sativa* L.), and tomato (*Solanum lycopersicum* L.) (Shavrukov et al. 2017; Panda et al. 2021; Conti et al. 2023).

Many species of legumes (Leguminosae, Fabaceae) are important food crops capable of fixing atmospheric nitrogen through symbiosis with rhizobia and accumulating high levels of storage proteins in seeds. Representatives of this group possess unique developmental features and exhibit considerable adaptive potential to abiotic stresses (Dubey et al. 2020; Khatun et al. 2021). Nevertheless, despite extensive physiological and molecular research and a long history of cultivation, several aspects of their reproductive development under both optimal and stress conditions remain insufficiently understood. In yellow lupin (*Lupinus luteus* L.), premature and excessive flower abscission has been observed (Glazińska et al. 2017). This reproductive instability contributes to reduced yields. To explain this undesirable phenomenon, several hypotheses have been proposed, including insufficient pollination of cleistogamous flowers and differential phytohormonal regulation (Marciniak et al. 2018b).

Reproductive development is particularly vulnerable to water deficit. Although plants can partially compensate for drought during vegetative growth through various tolerance mechanisms, stress occurring during the generative phase often leads to irreversible alterations (Oguz et al. 2022). Among reproductive processes, anther development is especially sensitive to drought conditions. Numerous studies indicate that severe water deficit markedly disrupts anther and pollen development, while female organ development is comparatively less affected (Saini and Lalonde 1997; Yu et al. 2019). Such male-biased sensitivity frequently results in impaired fertilization and reduced seed set. Therefore, elucidating the mechanisms underlying drought-induced male sterility is of particular importance for improving reproductive stability and ensuring crop productivity under increasingly variable environmental conditions.

Stamen development is a well-studied biological process that begins with the formation of primordia and the differentiation of somatic and germinal cell layers (Sanders et al. 1999; Zhang et al. 2021). The early phase of stamen development, during which meiosis occurs, is followed by a late developmental phase, that consists of filament elongation, anther dehiscence, and senescence. In general, the anther has a bilateral structure with four locules where pollen matures. The tissues adjacent to each locule are the tapetum, middle layer (ML), endothecium and epidermis, while in the middle part of the anther, a vascular bundle surrounded by connective tissue is located. In most angiosperms, stamen development involves coordinated and synchronized interactions between sporophytic and gametophytic tissues. This culminates in the development of functional pollen, its release, and ultimately pollination, fertilization, and seed set and development (Scott et al. 2004; Marciniak and Przedniczek 2019).

Phytohormones are signal molecules generated within plants at extremely low concentrations, with the ability to regulate numerous processes. Many phytohormone-deficient and phytohormone-response mutants are male sterile and possess anthers that are late-dehiscent or indehiscent (Marciniak and Przedniczek 2019). The late stages of stamen development are primarily regulated by jasmonates (JAs) and auxins; auxins also control stamen initiation (Song et al. 2013). Gibberellins (GAs) principally stimulate filament elongation and encourage anther opening. During GA-dependent regulation of early stamen development, both the tapetum and developing pollen have been identified as primary targets (Plackett et al. 2011; Marciniak and Przedniczek 2019). The role of salicylic acid (SA) has not been exhaustively studied (Luo et al. 2022). Furthermore, due to intricate phytohormonal networks of mutual interactions, plants can effectively respond to various external factors. The role of phytohormones in fostering plant acclimatization to consistently fluctuating environmental conditions is reasonably well established (Wolters and Jurgens 2009; Fahad et al. 2015). JAs are associated with plant defense against biotic stresses, but their importance in abiotic stresses is increasingly becoming evident. In various organs (spear tips, leaves, roots, whole seedlings) of many plants (*Asparagus officinalis*, *Carica papaya*, *Pinus pinaster*, *O. sativa*), the concentration of JAs often increases following exposure to drought stress, but occasionally, the accumulation of this phytohormone is unstable, displaying a transient increase followed by a decrease (*Glycine max*) (Llanes et al. 2016). Nevertheless, it is unclear precisely how drought influences the biosynthesis, accumulation, localization, signaling, and transport of JAs, as well as the interaction between JAs and other phytohormones in the flowers and anthers of diverse species.

In this paper, morphological and histological analyses revealed how drought affects *L. luteus* anthers structure and dehiscence time, closely tied to male fertility. This stress-induced phenotypic changes prompted us to measure jasmonic acid (JA) concentrations in different post-meiotic phases of anther development, using liquid chromatography coupled with tandem mass spectrometry (LC–MS/MS). Then, we performed immunohistochemical (IHC) assays to confirm that the disturbances in JA levels in anthers growing under unfavorable environmental conditions correlate with spatial shifts in the phytohormone localization at the cellular and tissue levels. The research was supplemented by the determination of the transcriptional activity (qPCR) of selected genes representing key steps of JA biosynthesis and perception, including *LlAOS*, *LlAOC*, *LlOPR3*, and *LlCOI*. The next goal was to identify other phytohormones that function in a JA-dependent pathway. For this purpose, mefenamic acid (MEF, JA biosynthesis inhibitor) was applied to demonstrate how reduced JA levels in the post-anther dehiscence phase of *L. luteus* grown under well-watered conditions influence changes in the expression of genes linked to the metabolism, signaling, and transport of various phytohormones (RNA-Seq analyses). Additionally, by limiting JA biosynthesis, we assessed its broader role in regulating the mRNA content of genes associated with carbohydrate and lipid metabolism, amino acid transport, water and ion transport, and the organization of different cellular structures. Finally, we determined the levels of selected JA-cooperating phytohormones such as GAs (GA_3_, GA_7_), SA, and indole-3-acetic acid (IAA) during drought stress in anthers, and also in flowers and vegetative organs. Our findings enhance the existing understanding of the JA-dependent mechanism of late anther development in *L. luteus* induced by drought stress, a crucial aspect for future attempts to increase the drought-resistance, control the development of male reproductive organs, and increase yields under adverse environmental conditions.

## Results

### Morphological and histological changes of *L. luteus* anthers growing under drought stress conditions

In our previous studies, we established a detailed classification of inflorescence, flower, and anther development in *L. luteus* (Marciniak and Przedniczek 2020, 2021; Marciniak et al. 2024). Inflorescence and flower development were divided into 10 phases (I1-I10 and F1-F10, respectively), whereas late anther development was classified into six morphologically and cytologically distinct stages (LAD1-LAD6). Although each LAD stage is correlated with a specific flower developmental phase, the classification is based on characteristic anatomical and cellular features of the anther during dehiscence and senescence. LAD1, occurring during I/F1-2, corresponds to the late post-meiotic stage of anther development. At this stage, the anther is tetrasporangiate and consists of four separate locules surrounded by differentiated wall layers, including the epidermis, endothecium with secondary wall thickening, ML, and remnants of the tapetum. The stomium region is already formed, and the septum connecting adjacent locules is still present, although the first signs of septum degeneration are visible. LAD2, occurring during I/F3-4, represents the active dehiscence stage. Progressive degeneration of septum cells followed by rupture of stomium cells leads to fusion of adjacent locules into a bilocular anther and finally to anther opening and pollen release. LAD3 (I/F5) is characterized by post-dehiscence degeneration of anther tissues. During this stage, progressive shrinkage and cellular degeneration of the anther are observed. During LAD4 (I/F6–7), the septum remnants become almost invisible, and the cells forming the anther wall degenerate, although the epidermis and endothecium are still visible. In the LAD5 (I/F8) and LAD6 (I/F9-10) phases, progressive collapse, shrinkage, and senescence of the entire anther structure occur, ultimately leading to complete degeneration of anther tissues. Thus, the LAD classification reflects defined developmental and structural states of the anther rather than merely chronological stages associated with flower development. Under drought stress conditions, the nomenclature of LAD stages was maintained according to the corresponding flower developmental phase; however, the anatomical progression of drought-treated anthers was frequently delayed compared with well-watered plants.

**Fig. 1:**
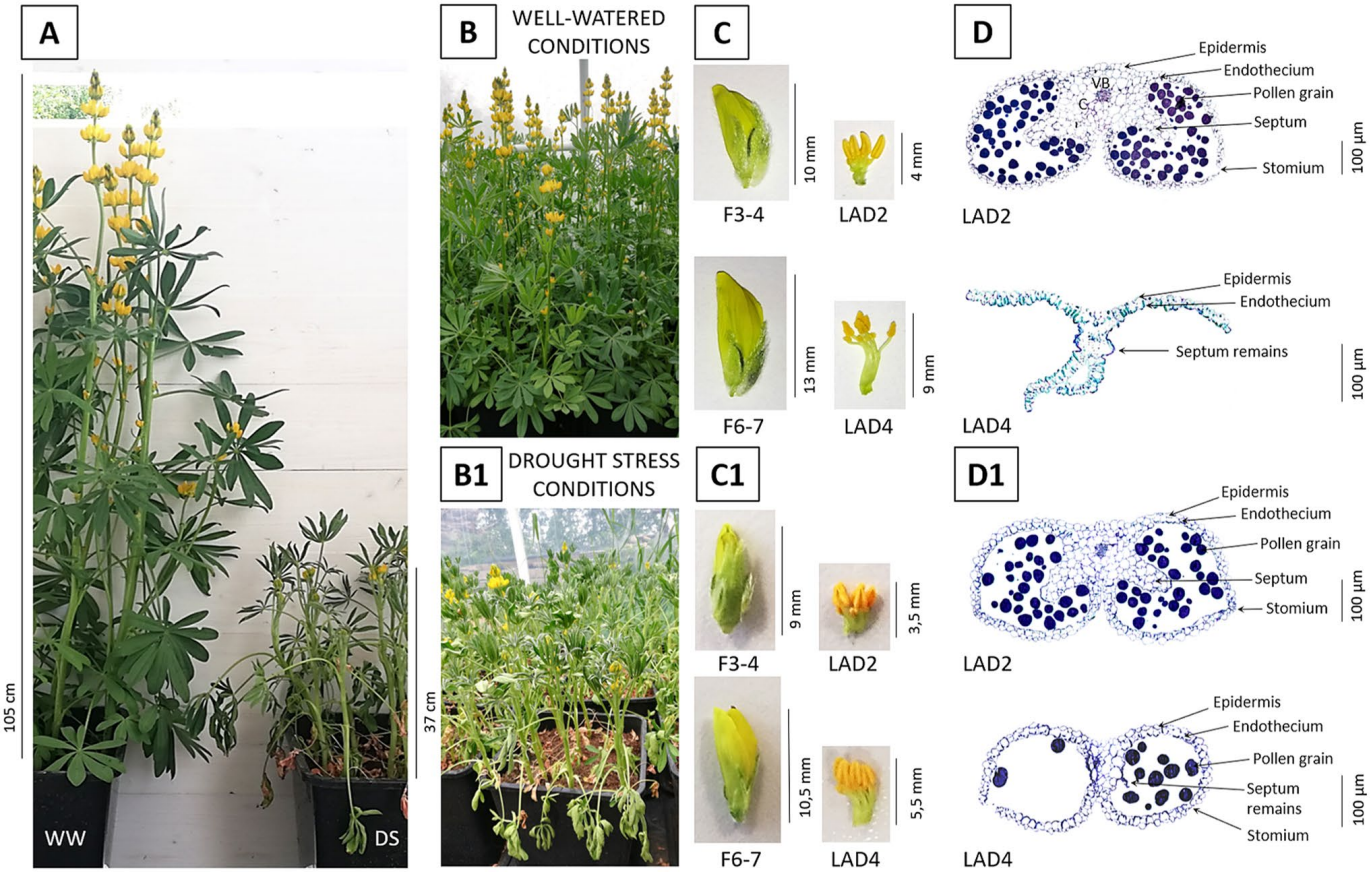
Morphological and histological changes during the whole plants, flowers, and anthers development in yellow lupin (*Lupinus luteus* L.) growing under drought stress conditions **(A, B1-D1)** compared to well-watered conditions **(A-D)**. Cross-sections were stained with toluidine blue and anthers were photographed by light microscopy. The developmental phases of flowers (F3-4, F6-7) and anthers (LAD2, LAD4) were selected acc. to Marciniak and Przedniczek 2020, 2021; Marciniak et al. 2024. C: connective, DS: drought stress conditions, F: flower, LAD: late anther development, VB: vascular bundle, WW: well-watered conditions

In this study, we determined the effect of drought on the development of anthers, flowers, and whole *L. luteus* plants using morphological and histological analyses. At the morphological level of the entire plant, stress conditions significantly shortened the stem (drought: approx. 37 cm, well-watered: approx. 105 cm on average) and inhibited the development of upper and lower leaves, roots, and inflorescence (Fig. 1A, B1) compared to well-watered conditions (Fig. 1A, B). Even under water deficit, plants were still able to flower (Fig. 1A, B1).

In the F3-4 and F6-7 developmental phases, flowers exposed to drought stress were smaller (9 mm and 10.5 mm on average, respectively) (Fig. 1C1) than those developed under well-watered conditions (10 mm and 13 mm on average, respectively) (Fig. 1C). Differences between control and drought-treated plants were also evident during anther development (Fig. 1C, C1). Under drought stress, anthers assigned to the LAD4 stage based on flower developmental phase exhibited morphological characteristics resembling control LAD2 anthers. Their surface remained relatively smooth and frequently appeared unopened (Fig. 1C1), whereas LAD4 anthers developing under well-watered conditions displayed a rough and uneven surface associated with tissue degeneration (Fig. 1C). The average length of whole stamens during the LAD4 and LAD2 phases under drought stress was 5.5 mm and 3.5 mm, respectively (Fig. 1C1). By contrast, stamens developing under well-watered conditions reached approximately 9 mm in LAD4 and 4 mm in LAD2 (Fig. 1C), indicating inhibited stamen elongation under drought conditions.

Histological analyses confirmed that anther development under drought stress proceeded similarly to control conditions up to the LAD2 stage (Fig. 1D1, D). In both conditions, progressive septum degeneration, stomium formation, and differentiation of anther wall layers were observed. Additionally, pollen grains were present in comparable quantities. In drought-treated anthers, no persistent tapetum or tapetum remnants were detected during LAD2, despite reports from other species indicating drought-dependent inhibition of tapetum PCD and associated tapetal hypertrophy (Zhang et al. 2021). The only visible difference at this stage was the slightly smaller size of drought-treated anthers compared with well-watered controls. The most pronounced drought-induced alterations were observed during the LAD4 stage (Fig. 1D1). In contrast to well-watered plants, in which LAD4 represented a senescence stage with strongly degenerated anther tissues and released pollen grains, drought-treated LAD4 anthers frequently remained unopened or displayed only initial signs of dehiscence. Although substantial septum degeneration was visible, the anther wall cells were less degraded than in control LAD4 anthers, indicating delayed developmental progression under drought stress. Additionally, the number of pollen grains in drought-treated LAD4 anthers was lower than in drought-treated LAD2 anthers.

### Drought stress disrupts the concentration of JA during *L. luteus* anther development

The drought-induced abnormal anther development in *L. luteus* might be due to changes in JA concentrations. From our previous paper, it follows that this phytohormone plays an important role in the development of male reproductive organs (Marciniak et al. 2024). This formed the basis for examining the endogenous JA levels in anthers maturing during drought-stress conditions. For a more comprehensive overview, we also determined the JA concentrations in flowers and in vegetative organs (leaves, stems, roots).

**Fig. 2:**
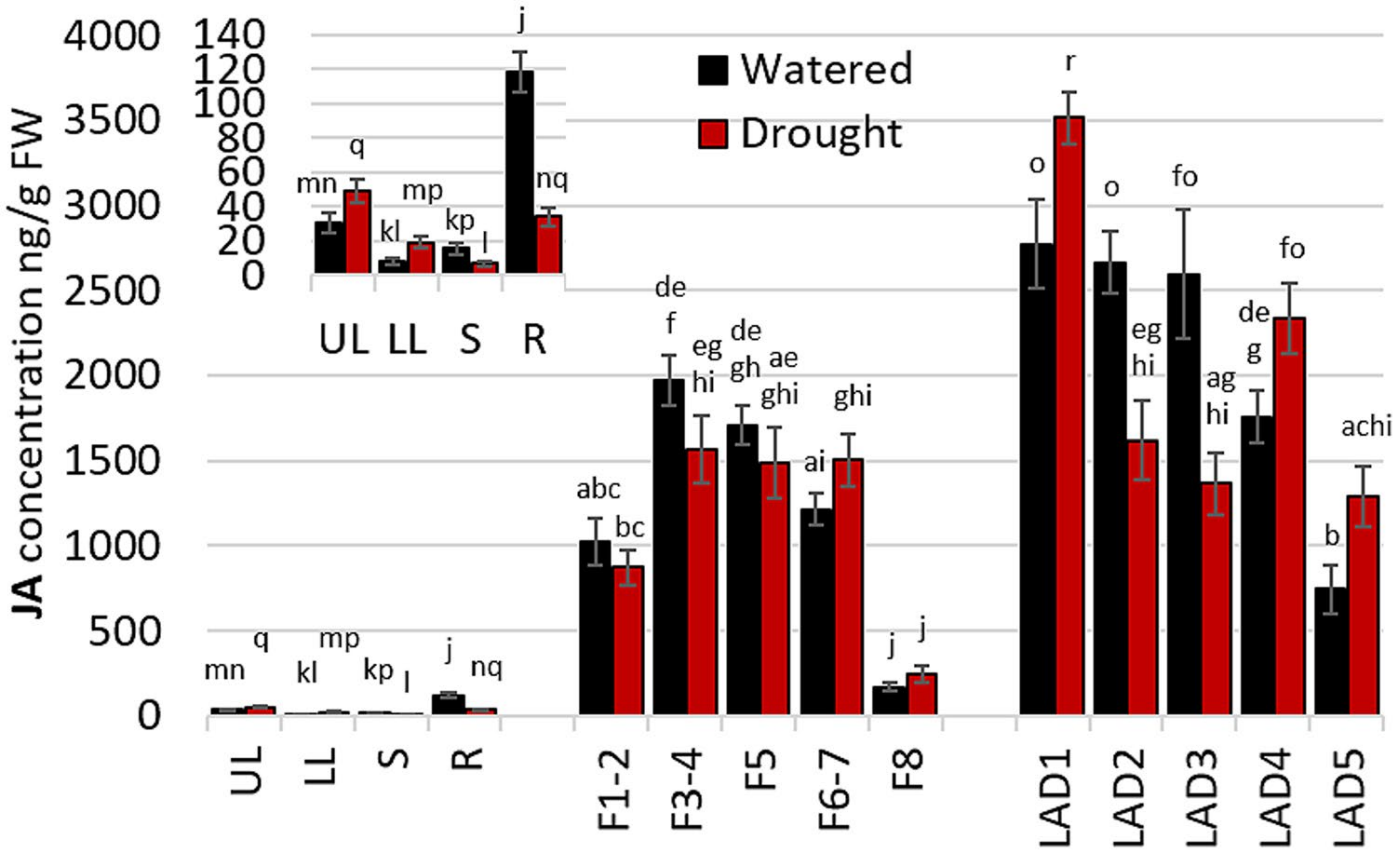
Jasmonic acid (JA) concentrations in the reproductive and vegetative organs of yellow lupin (*Lupinus luteus* L.) growing under drought stress and well-watered conditions. Data are the mean ± SD of three biological replicates, each with two technical replicates. Statistical differences were assessed using Welch’s ANOVA (*p* < 0.01), followed by the Games-Howell test (*p* < 0.05). Different letters indicate significant differences between groups (Compact Letter Display). F: flowers, LAD: late anther development, LL: lower leaves, UL: upper leaves, R: roots, S: stems

Our results demonstrated that drought stress altered the phytohormonal balance during the late phases of *L. luteus* anther development. JA accumulation patterns were disturbed and reversed under drought conditions: JA levels were lower during the LAD2-3 phases, in which they were normally high under well-watered conditions, whereas higher levels were detected during the LAD4-5 phases, in which JA content typically declined (Fig. 2).

Under well-watered conditions, the JA concentrations in *L. luteus* flowers were similar to those in anthers (excluding F1-2), but remained at lower levels. The effects of drought were negligible in all phases (Fig. 2). Stress conditions increased JA levels in the upper and lower leaves but decreased them in the stems. Interestingly, roots developing under drought showed a substantial decrease in JA concentrations (Fig. 2).

### Alterations in JA localization during specific phases of anther development under drought stress correlate with fluctuations in its concentration

**Fig. 3:**
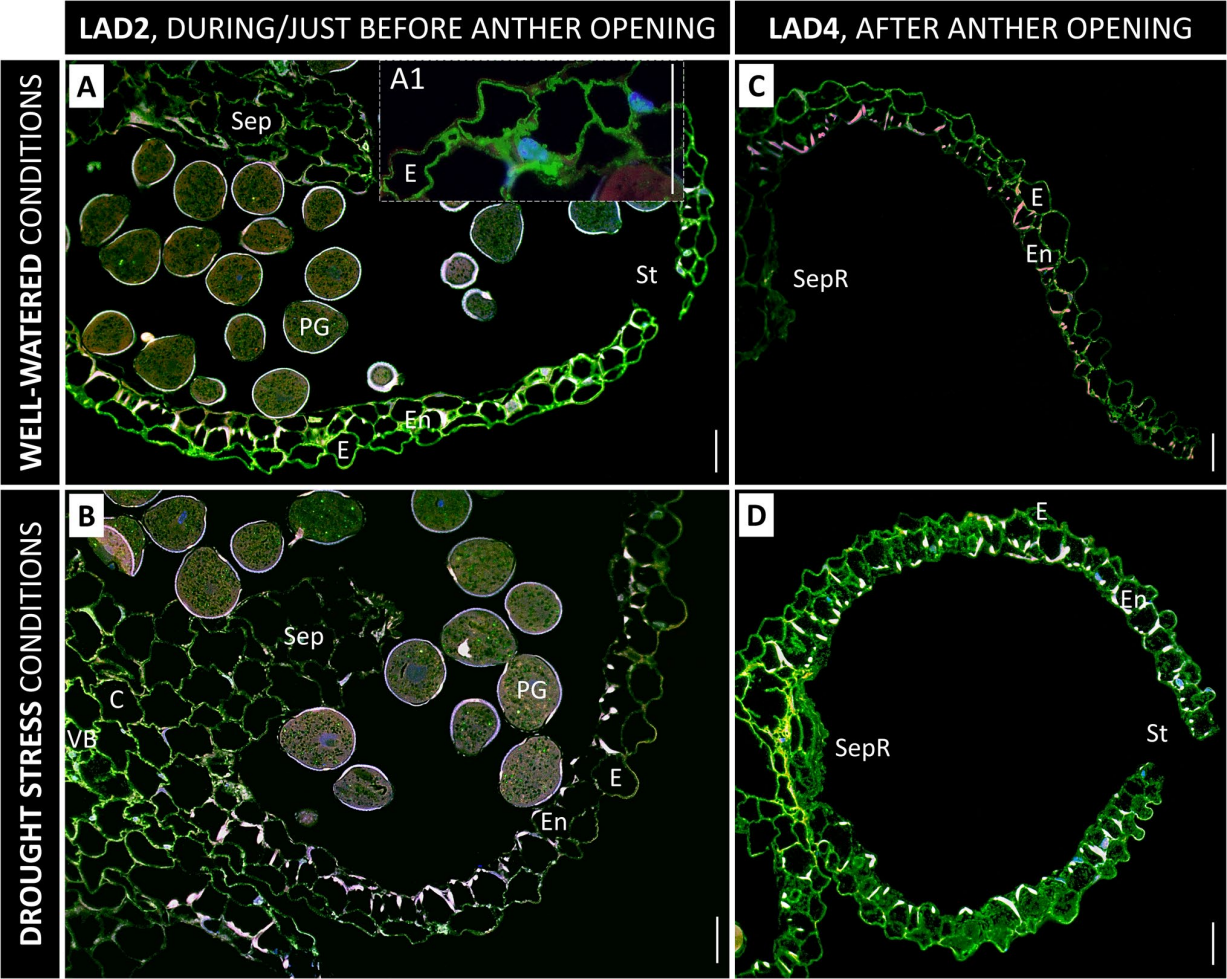
Jasmonic acid (JA) localization during yellow lupin (*Lupinus luteus* L.) anther development in the LAD2 phase **(A-B)**, and in the LAD4 **(C-D)**, both in well-watered and drought stress conditions. The square marked with a white dashed line represents stomium region before opening **(A1)**. The green fluorescence signal indicates JA accumulation, and the blue signal indicates DAPI-stained cell nuclei. Autofluorescence of compounds after excitation with UV light (blue autofluorescence) and green light (red autofluorescence) is visible. Other colors were created by overlapping green, blue or red (ImageJ). C: connective, E: epidermis, En: endothecium, LAD: late anther development, PG: pollen grain, Sep: septum, SepR: septum remains, St: stomium, VB: vascular bundle. Scale bars 20 μm (A-D) and 25 μm (A1)

We investigated JA localization in anthers growing under drought stress in two development phases—LAD2, where the JA concentration apparently diminished without noticeable structural alterations in anthers, and LAD4, where the JA level increased compared to well-watered conditions and structural disorders were observable. Our findings suggest that fluctuations in JA concentrations during these developmental phases under differing conditions (Fig. 2) strongly correlate with alterations in cellular and tissue localization (Fig. 3). In the LAD2 phase under well-watered conditions (Fig. 3A), a green fluorescence signal indicating the presence of JA was accumulated in the septum and particular cells of the anther wall, primarily in the epidermis. The signal was also visible in other cells of the anther wall, especially the closer these cells were to the stomium. JA was also detected in the stomium itself (Fig. 3A1), presumably facilitating anther opening. In the LAD2 phase under drought conditions (Fig. 3B), JA signal was present in the same cell types but its visibility was lower. This relationship was observed primarily in epidermis and other cells forming the anther wall, because the JA signal in the septum and the area of the vascular bundle was quite clearly visible.

After the anthers were opened, pollen grains were released, and progressing degeneration and shrinkage of cells were noticeable during the LAD4 phase under well-watered conditions (Fig. 3C), JA signal was detected in the particular cells that form remnants of the anther wall, although it was weaker than in the cells in the LAD2 phase (Fig. 3A). In turn, during the LAD4 phase of anthers developing under drought conditions (Fig. 3D), a strong green fluorescence signal was found within the cells forming the anther walls. The JA was located close to the cell walls and also was dispersed throughout the cytoplasm.

### Drought stress affects the expression of JA biosynthesis and perception genes in anthers and other organs of *L. luteus* depending on their specific developmental phase

**Fig. 4:**
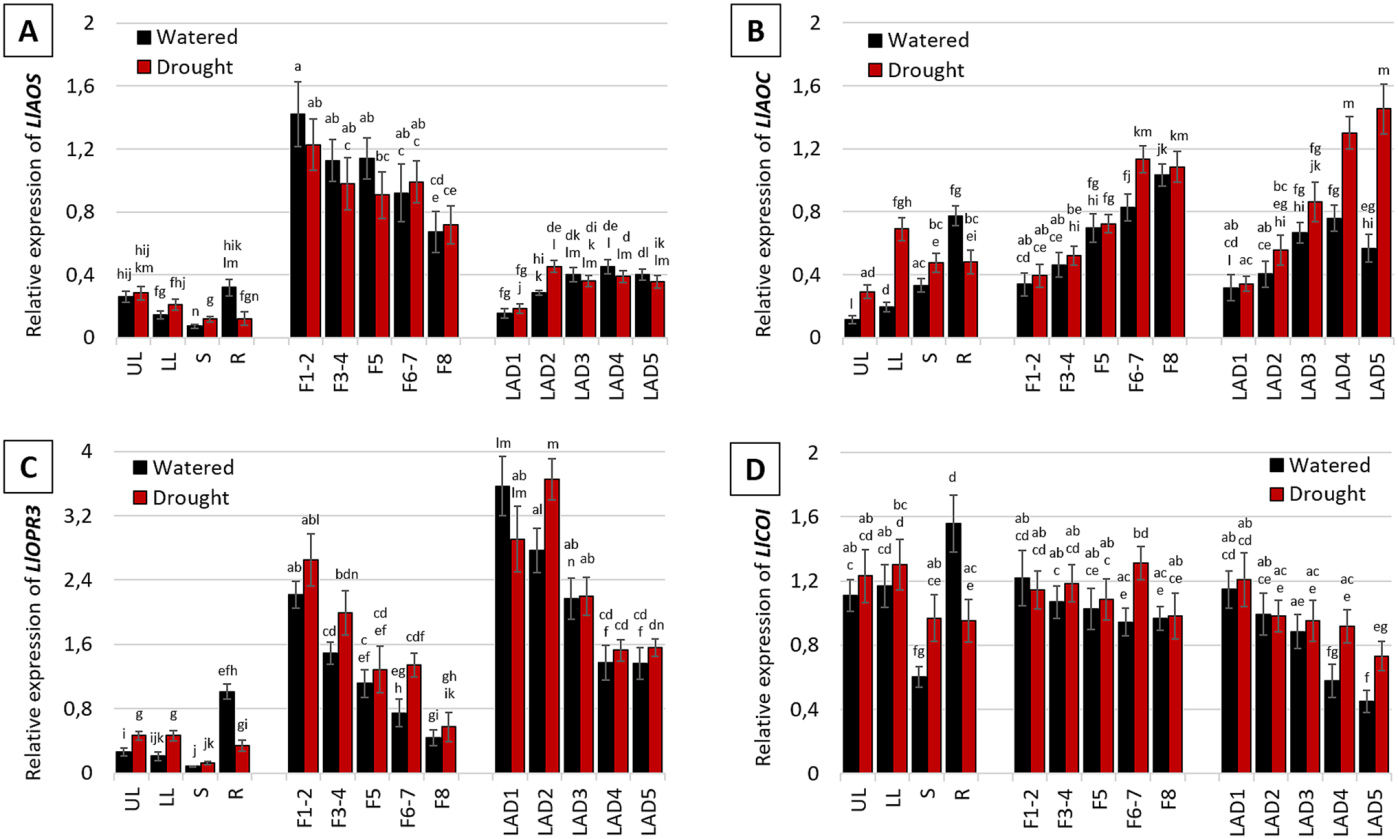
Relative expression of *LlAOS* (*ALLENE OXIDE SYNTHASE*) **(A)**, *LlAOC* (*ALLENE OXIDE CYCLASE*) **(B)**, *LlOPR3* (*OPDA REDUCTASE 3*) **(C)**, and *LlCOI1* (*CORONATINE INSENSITIVE 1*) **(D)** genes involved in JA biosynthesis and perception in reproductive and vegetative organs of yellow lupin (*Lupinus luteus* L.) growing under well-watered and drought stress conditions. *ACTIN* (*LlACT*) was used as an internal control. Data are the mean ± SD of three biological replicates, each with two technical replicates. Statistical differences were assessed using Welch’s ANOVA (*p* < 0.01), followed by the Games-Howell test (*p* < 0.05). Different letters indicate significant differences between groups (Compact Letter Display). F: flowers, LAD: late anther development, LL: lower leaves, UL: upper leaves, R: roots, S: stems

To ascertain the effects of drought stress on the transcriptional activity of genes associated with JA biosynthesis and perception, we conducted qPCR analyses for *LlAOS*, *LLAOC*, *LlOPR3*, and *LlCOI* in different stages of *L. luteus* anther development. We also included flowers and vegetative organs such as upper leaves, lower leaves, stems, and roots for a more comprehensive view (Fig. 4). For *LlAOS* (Fig. 4A) and *LlOPR3* (Fig. 4C), drought did not significantly affect their expression during anther dehiscence and senescence, aside from the LAD2 phase, which exhibited an increase in these genes’ transcript numbers. The expression of the two other genes, *LlAOC* (Fig. 4B) and *LlCOI* (Fig. 4D), remained unchanged in the LAD1-3 phases under stress conditions but increased strongly in the LAD4-5 phases.

The transcriptional activity of selected genes in flowers during drought did not vary from that under well-watered conditions. Nonetheless, an increase in the expression of *LlAOC*, *LlOPR3*, and *LlCOI1* (Fig. 4B-D) was noticed in the F6-7 phase. For *L. luteus* roots grown under drought stress, the expression of *LlAOS*, *LlAOC*, *LlOPR3*, and *LlCOI1* (Fig. 4A-D) decreased compared to optimal conditions. In both upper and lower leaves, *LlAOC* and *LlOPR3* mRNAs increased (Fig. 4B, C), while the amount of *LlAOS* and *LlCOI* remained unchanged (Fig. 4A, D). In terms of the stem, the most notable increase in transcript abundance was observed for *LlCOI*, followed by *LlAOS* (Fig. 4D, A).

### The limited JA biosynthesis triggers specific changes in the expression of genes related to different phytohormones in post-dehiscence anthers of *L. luteus*

The JA biosynthesis inhibitor MEF was intentionally applied during the LAD3 phase because this developmental stage is characterized by naturally high JA levels under well-watered conditions, followed by a rapid decline during the subsequent LAD4 phase. Our objective was to experimentally induce a premature reduction in JA content at the LAD3 stage in order to identify downstream molecular processes and, primarily, candidate phytohormonal pathways regulated in a JA-dependent manner. This transcriptomic experiment was designed as an exploratory screening approach to generate biological hypotheses for subsequent physiological and biochemical verification under drought conditions. Additionally, the transition between LAD3 and LAD4 appears to be an important developmental window, because this is also the stage at which drought stress causes the strongest disturbance in JA homeostasis. For this reason, we performed the transcriptomic experiment under well-watered conditions following MEF treatment rather than directly under drought stress. Drought induces broad and complex molecular responses involving numerous stress-related pathways, making it difficult to distinguish processes specifically associated with JA-dependent regulation. In contrast, the MEF-based experimental system enabled us to selectively identify downstream molecular and phytohormonal pathways associated with reduced JA biosynthesis, without the confounding effects of global drought-induced transcriptomic reprogramming.

Our GSEA revealed that MEF treatment impacts the transcriptional activity of genes associated with GA and SA metabolism (Table 1A, B) and auxin signaling and transport (Table 1C, D). Among the DEGs linked with GA inactivation and biosynthesis, we identified genes annotated as GIBBERELLIN 2-β-DIOXYGENASES (GA2oxs, connected with phytohormone inactivation, upregulated) and *ent*-KAURENE SYNTHASE A/*ent*-COPALYL DIPHOSPHATE SYNTHASE (KSA/CPS, connected with phytohormone biosynthesis, downregulated) (Table 1A). Additionally, upregulated DEGs encoding MYB62/MYB2 transcription factors, previously linked to GA-related regulation and phytohormonal stress responses (Devaiah et al. 2009), were identified. The reduction in JA biosynthesis led to an increase in the expression levels of genes annotated as DOWNY MILDEW RESISTANCE 6 (DMR6) and DMR6-LIKE OXYGENASE 1 (DLO1) (Table 1B), which are associated with the negative feedback regulation of SA levels (Zeilmaker et al. 2015). We identified also 17 upregulated genes related to the auxin-activated signaling pathway (Table 1C), and 3 upregulated genes linked with auxin transmembrane transporter activity (Table 1D). These DEGs have been annotated as: AUXIN RESPONSE FACTOR 4 (ARFD), various SAURs, several PIN-LIKES (PILSs) and ABC TRANSPORTERs B FAMILY MEMBER (ABCBs), NACs (NAC domain-containing proteins), and transferases – GLUTATHIONE S-TRANSFERASES (GSTs) and N-ACETYLTRANSFERASE HOOKLESS1 (HLS1).

**Table 1:** Differentially expressed genes (DEGs) associated with significantly overrepresented (*p* < 0.05) Gene Ontology (GO) terms related to phytohormone metabolism, signaling, and transport in the MEF-Control comparison. GO significance refers to the enriched GO terms, whereas Log2FC and PPEE values correspond to the differential expression statistics for the individual genes

(A) Gibberellin biosynthetic process (GO:0009686)					
Gene Id	No. of Unigenes	Annotation Id	Blast Annotation Full Name	PPEE	Log_2_(FC)
TRINITY_DN11825_c3_g1	4	MYB62_ARATH	Transcription factor MYB62 {ECO:0000303|PubMed:9,839,469}	1.9149E-07	4.137967091
TRINITY_DN11825_c3_g4	2	MYB2_ORYSJ	Transcription factor MYB2 {ECO:0000305}	6.1522E-05	3.410630658
		MYB62_ARATH	Transcription factor MYB62 {ECO:0000303|PubMed:9,839,469}		
TRINITY_DN12295_c0_g1	2	G2OX_PHACN	Gibberellin 2-beta-dioxygenase	2.6381E-05	3.144534116
TRINITY_DN12295_c1_g2	6	G2OX1_PEA	Gibberellin 2-beta-dioxygenase 1	9.156E-05	2.698729519
TRINITY_DN5885_c0_g1	3	KSA_PEA	*Ent*-copalyl diphosphate synthase, chloroplastic	0.03296054	− 2.22648558

(B) Salicylic acid catabolic process (GO:0046244)					
Gene Id	No. of Unigenes	Annotation Id	Blast Annotation Full Name	PPEE	Log_2_(FC)
TRINITY_DN11477_c5_g1	4	DMR6_ARATH	Protein DOWNY MILDEW RESISTANCE 6 {ECO:0000303|PubMed:15,986,928}	0.02151133	2.616395908
		F6H1_ARATH	Feruloyl CoA ortho-hydroxylase 1		
TRINITY_DN12619_c0_g4	5	DMR6_ARATH	Protein DOWNY MILDEW RESISTANCE 6 {ECO:0000303|PubMed:15,986,928}	0.00303228	2.317367782
TRINITY_DN11626_c3_g6	2	DIOX5_ARATH	Probable 2-oxoglutarate-dependent dioxygenase At5g05600 {ECO:0000305}	0.02330885	2.061925279
		DLO1_ARATH	Protein DMR6-LIKE OXYGENASE 1 {ECO:0000303|PubMed:25,376,907}		
		FL3H_PETCR	Flavanone 3-dioxygenase		

(C) Auxin-activated signaling pathway (GO:0009734)					
Gene Id	No. of Unigenes	Annotation Id	Blast Annotation Full Name	PPEE	Log_2_(FC)
TRINITY_DN17764_c1_g3	4	ARFD_ARATH	Auxin response factor 4	2.1137E-10	5.287167042
TRINITY_DN17047_c4_g2	2	GSTX1_TOBAC	Probable glutathione S-transferase	1.068E-13	5.248279902
		GSTX2_TOBAC	Probable glutathione S-transferase		
TRINITY_DN7896_c0_g1	2	GSTXA_TOBAC	Probable glutathione S-transferase parA	3.5527E-15	5.145950702
TRINITY_DN13747_c2_g6	2	AB11B_ARATH	ABC transporter B family member 11	1.9184E-05	4.853093359
		AB4B_ARATH	ABC transporter B family member 4		
TRINITY_DN16000_c0_g5	1	SAU32_ARATH	Auxin-responsive protein SAUR32 {ECO:0000305}	0.0102179	3.198485685
TRINITY_DN17764_c1_g6	2	ARFD_ARATH	Auxin response factor 4	0.00389272	3.176893797
TRINITY_DN11798_c2_g3	2	NAC22_ARATH	NAC domain-containing protein 21/22	0.00013634	2.863497028
		NAC31_ARATH	Protein CUP-SHAPED COTYLEDON 3		
TRINITY_DN15861_c0_g1	2	GSTX4_TOBAC	Probable glutathione S-transferase	0.00276106	2.858376944
TRINITY_DN10546_c2_g2	4	HLS1L_ARATH	Probable N-acetyltransferase HLS1-like	0.00023011	2.647284538
		HLS1_ARATH	Probable N-acetyltransferase HLS1		
TRINITY_DN16329_c4_g3	2	ARFD_ARATH	Auxin response factor 4	0.00154887	2.54539596
TRINITY_DN13112_c0_g1	10	GSTUJ_ARATH	Glutathione S-transferase U19	0.00115598	2.48129443
		GSTX3_SOYBN	Glutathione S-transferase 3		
		GSTX4_TOBAC	Probable glutathione S-transferase		
TRINITY_DN12449_c1_g4	24	PILS7_ARATH	Protein PIN-LIKES 7 {ECO:0000303|PubMed:22,504,182}	0.00096415	2.460489495
TRINITY_DN11798_c2_g1	5	NAC22_ARATH	NAC domain-containing protein 21/22	0.0011658	2.426081752
		NAC98_ARATH	Protein CUP-SHAPED COTYLEDON 2		
TRINITY_DN12449_c1_g2	8	PILS5_ARATH	Protein PIN-LIKES 5 {ECO:0000303|PubMed:22,504,182}	0.00207893	2.357492123
		PILS7_ARATH	Protein PIN-LIKES 7 {ECO:0000303|PubMed:22,504,182}		
TRINITY_DN12969_c1_g5	8	IAA26_ARATH	Auxin-responsive protein IAA26	0.00699899	2.225142152
TRINITY_DN10379_c5_g1	9	VAB_ARATH	VAN3-binding protein {ECO:0000303|PubMed:19,363,154}	0.00767535	2.204019844
TRINITY_DN27094_c0_g1	1	SAU50_ARATH	Auxin-responsive protein SAUR50 {ECO:0000303|PubMed:12,036,261}	0.02794353	2.065135802

(D) Auxin transmembrane transporter activity (GO:0080161)					
Gene Id	No. of Unigenes	Annotation Id	Blast Annotation Full Name	PPEE	Log_2_(FC)
TRINITY_DN13747_c2_g6	2	AB11B_ARATH	ABC transporter B family member 11	1.9184E-05	4.853093359
		AB4B_ARATH	ABC transporter B family member 4		
TRINITY_DN13747_c2_g3	1	AB11B_ARATH	ABC transporter B family member 11	0.00027267	4.011068817
TRINITY_DN12449_c1_g2	8	PILS5_ARATH	Protein PIN-LIKES 5 {ECO:0000303|PubMed:22,504,182}	0.00207893	2.357492123
		PILS7_ARATH	Protein PIN-LIKES 7 {ECO:0000303|PubMed:22,504,182}		

Gene annotations were obtained from the Swiss-Prot database using BlastP and BlastX. *FC* fold change; *PPEE* Posterior Probability of Equal Expression; *ARATH* Arabidopsis thaliana; *ORYSJ* Oryza sativa; *PEA* Pisum sativum; *PETCR* Petroselinum crispum; *PHACN* Phaseolus coccineus; *SOYBN Glycine max*; *TOBAC* Nicotiana

In the MEF-Control comparison, GO enrichment analyses also revealed significant differences in gene expression related to critical biological processes, such as metabolism, transport, and homeostasis of organic (carbohydrates, lipids, amino acids) and inorganic (water, ions, micro- and macro-elements) substances, as well as the organization of various structures (cell walls, channels) in *L. luteus* anthers grown under well-watered conditions (Fig. S1-S6). We also uncovered a large network of interconnections among overrepresented GO terms related to: biotic factors (fungus, oomycetes), phytohormones (JAs, GAs, auxins), redox reactions/antioxidants (L-ascorbic acid), and ion transport (cadmium) (Fig. S7). A similar network included GO terms associated with: rhythmic processes, photoperiodism, phytohormones (auxins), polysaccharides, and water deprivation (Fig. S8). The vast majority of DEGs assigned to important GO terms were upregulated in both networks. Detailed analyses are presented in the supplementary materials.

### Drought stress alters the concentrations of JA-interacting phytohormones during late anther development in *L. luteus*

**Fig. 5:**
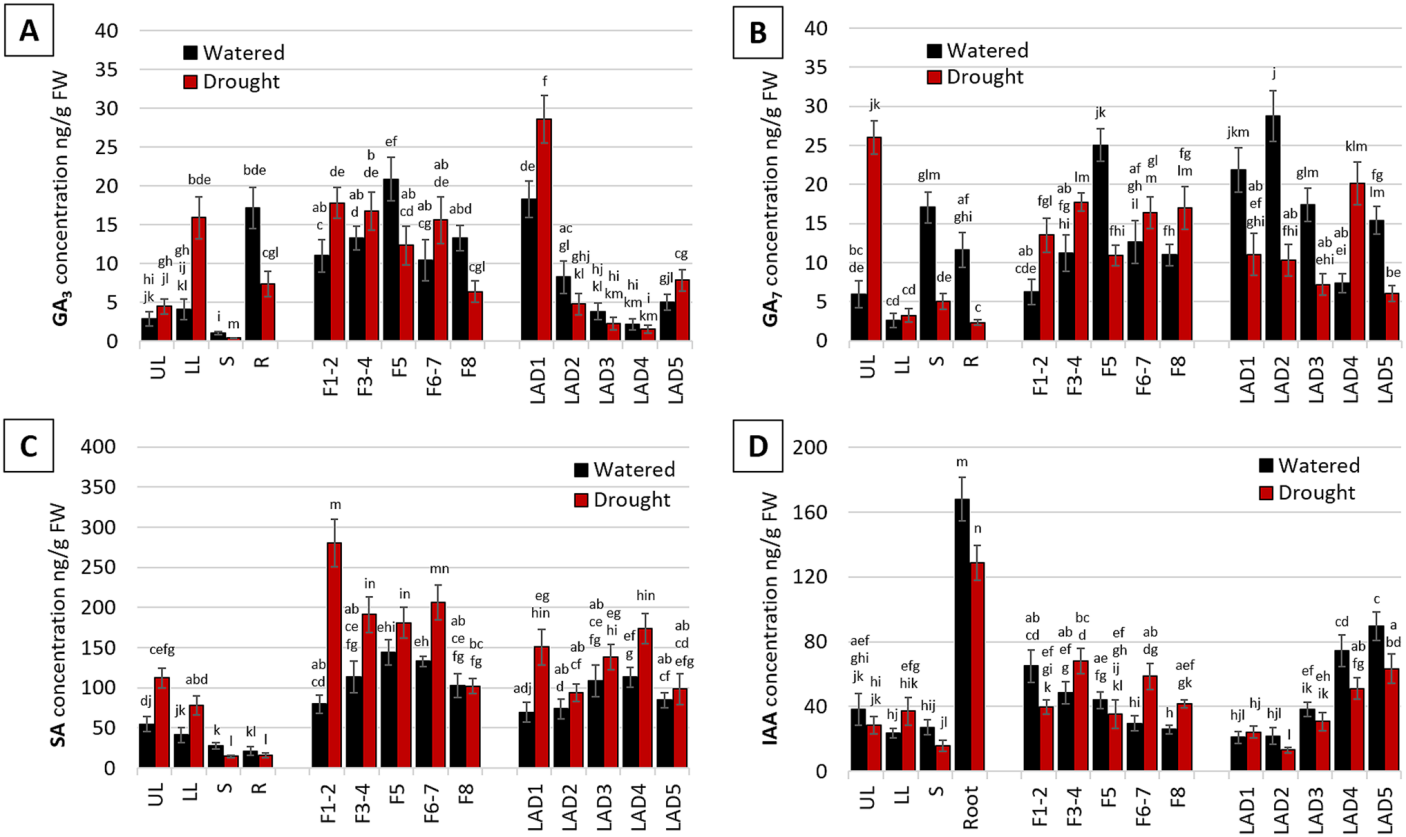
The concentrations of gibberellic acid (GA_3_) **(A)**, GA_7_ **(B)**, salicylic acid (SA) **(C)**, and indole-3-acetic acid (IAA) **(D)** in reproductive and vegetative organs of yellow lupin (*Lupinus luteus* L.) growing under drought stress and well-watered conditions. Data are the mean ± SD of three biological replicates, each with two technical replicates. Statistical differences were assessed using Welch’s ANOVA (*p* < 0.01), followed by the Games–Howell test (*p* < 0.05). Different letters indicate significant differences between groups (Compact Letter Display). F: flowers, LAD: late anther development, LL: lower leaves, UL: upper leaves, R: roots, S: stems

In the preceding section, we demonstrated that various phytohormones are part of a complex, JA-dependent network that regulates *L. luteus* anther development. Now, we measured the endogenous levels of selected phytohormones (GAs, SA, IAA) during anther maturation in different environmental conditions. Additionally, we determined the phytohormone concentrations in flowers and in vegetative organs (Fig. 5).

Similar to JA under well-watered conditions, the phytohormones that exhibited higher levels during anther dehiscence (LAD1-3) and lower during anther senescence (LAD4-5) were GAs—GA_3_ and GA_7_ (Fig. 5A, B). The highest and average concentration of GA_3_ was 18 ng/g FW in the LAD1 phase (Fig. 5A), and GA_7_ – was 29 ng/g FW in the LAD2 phase (Fig. 5B). Conversely, the IAA levels increased with the progressive aging of anthers. In the last two phases of anther development (LAD4-5), the IAA concentrations were on average 75–90 ng/g FW (Fig. 5D). The maximum SA levels mainly occurred around the LAD3 and LAD4 phases, with average concentrations of 108–113 ng/g FW (Fig. 5C). However, it was found that JA has definitely the highest levels among all tested phytohormones (averaging 2596–2872 ng/g FW in the LAD1-3 phases) (Fig. 2). In turn, drought stress changed the balance of phytohormones during the late phases of *L. luteus* anther development. The phases where drought increased concentrations were LAD1 and LAD4 in the case of phytohormones such as SA and GAs (GA_3_ in the LAD1 phase and GA_7_ in the LAD4 phase) (Fig. 5A-C). In the remaining LAD phases, drought did not induce such changes; the stress’s impact was insignificant (GA_3_, SA) (Fig. 5A, C) or reduced the amount of phytohormone (GA_7_) (Fig. 5B). The IAA concentration remained unchanged during anther dehiscence under drought stress and decreased during senescence (Fig. 5D).

The SA concentration increased in subsequent stages of flower development under optimal conditions, peaking in the F5 phase, and then decreased (Fig. 5C). Drought conditions prompted elevated levels of SA in F1-2, F3-4, and F6-7 phases, with the most notable increase occurring at the beginning (Fig. 5C). A similar correlation as with SA under well-watered conditions was noted for GA_3_ and GA_7_, showing the highest concentration definitely in the F5 phase (Fig. 5A-B). Interestingly, drought stress led to a slight increase in the concentration of GAs, primarily in the F1-2 phase; a significant decrease in the content of both GAs in the F5 phase (as well as in the F8 phase for GA_3_) stands out (Fig. 5A-B). With IAA under optimal conditions, the level peaked in the F1-2 phase, then declined, whereas the effect of drought was phase-specific (Fig. 5D). In the leaves, drought increased the levels of phytohormones like SA and GAs (GA_3_ in the lower leaves and GA_7_ in the upper leaves) (Fig. 5A-C), but had no significant effect on IAA concentration (Fig. 5D). In the stem, stress conditions decreased the content of SA, GA_3_, and GA_7_ (Fig. 5A-C), with no tangible effect on the IAA concentration (Fig. 5D). Interestingly, under stress conditions, a substantial reduction was reported in phytohormone concentrations, except SA, in roots** (**Fig. 5A-D).

## Discussion

Morphological and histological analyses of *L. luteus* anthers exposed to drought stress revealed that their development was typical up to the LAD2 phase, while changes were noticeable in the LAD4 phase. The differences concerned: (1) the anther wall cells, which were less degenerated than the same cells in anthers developing under well-watered conditions, and (2) the pollen chambers, which were still unopened or the process was just beginning. However, in the LAD4 phase, the septum cells were degenerated. These results demonstrated that *L. luteus* anthers growing under stress conditions exhibit a late-dehiscence phenotype that prevents the release of pollen grains, ultimately reducing their number. For other crops like *O. sativa* and *S. lycopersicum*, drought-induced infertility or sterility often results from abnormal development of male reproductive organs, which includes late-dehiscent or nondehiscent pollen chambers. This leads to impaired pollination, fertilization, and subsequent flower abortion and yield reduction (Jin et al. 2013; Lamin-Samu et al. 2021; Zhang et al. 2021).

Erroneous anther development in unfavorable environments is usually caused by phytohormonal imbalances in many plants (Yu et al. 2019). Therefore, considering the importance of JAs for proper *L. luteus* anther dehiscence and senescence (Marciniak et al. 2024), we investigated whether drought stress affects endogenous JA levels during late anther development. Our results showed that stress conditions disrupted and reversed JA accumulation patterns: JA levels were lower in drought-treated anthers during the LAD2–3 phases, in which they were normally high under well-watered conditions, but higher during the LAD4–5 phases, in which they typically declined. This imbalance may contribute to male fertility disorders, thereby indirectly affecting yield quantity and quality. In other plants, such as rice and *A. thaliana*, the homeostasis of the internal environment is also disturbed in anthers exposed to drought, which generally includes an increase in JA content (Yu et al. 2019).

Next, we performed JA immunolocalization in *L. luteus* anthers growing under different conditions. The key finding was that during drought stress, the green fluorescence signal indicating JA accumulation was lower in specific anther cells at the LAD2 phase, but significantly higher at the LAD4 phase, compared with well-watered conditions. These drought-induced temporal and spatial alterations in JA accumulation are likely involved in the substantial delay in pollen chamber opening in *L. luteus*. Consistently, drought also affected the expression of JA biosynthesis and perception genes in a developmentally dependent manner during anther dehiscence and senescence. In particular, the transcriptional activity of *LlAOC* and *LlCOI* increased in the LAD4 and LAD5 phases under water deficit. Therefore, the observed gene expression patterns were consistent with the findings obtained using analytical and immunohistochemical approaches. In summary, our results indicate that drought-induced, multilevel JA dysregulation, supported by temporal, spatial, and molecular evidence, coincides with phenotypic changes in *L. luteus* anthers. This suggests a link between JA imbalance and alterations in anther anatomy and function, although JAs likely act as part of a broader regulatory network involved in drought stress responses.

The use of MEF in stable, stress-free conditions of *L. luteus* anther development allowed us to select JA-dependent genes related to various phytohormones. Transcriptomic analyses revealed that specific changes occurred for mRNA content of genes associated with GA and SA metabolism, as well as auxin signaling and transport, suggesting a mutual interactions between phytohormones at the molecular level. Consistently, the limitation of JA biosynthesis may lead to GA inactivation and simultaneous inhibition of GA biosynthesis. In our previous work, we demonstrated that exogenous GA_3_ increases, and paclobutrazol (PAC, a GA biosynthesis inhibitor) decreases, the amount of JA at the right time and place during *L. luteus* anther development (Marciniak et al. 2024). This suggests the existence of a feedback loop regulating JA and GA levels that is likely important for proper *L. luteus* anther development. The increased expression of genes related to the negative feedback regulatory system for SA concentration suggests that this phytohormone may act similarly to JAs and GAs. In turn, absence of JAs following MEF application led to enhanced transcriptional activity of genes associated with signaling and transport of auxins. These transcriptomic results align with findings obtained by the chromatographic analyses, indicating that decreasing JA concentrations during the appropriate time of *L. luteus* anther development correlate with an increase in auxin levels, affirming the interdependencies between phytohormones. The restriction of JA biosynthesis following the opening of pollen chambers also affects the expression of genes associated with the metabolism, transport, and homeostasis of several organic and inorganic substances, as well as the organization of differing structures in *L. luteus* anthers growing under well-watered conditions. All these contribute to the proper development of male reproductive organs, and ultimately, to successful pollination, fertilization, and seed/pod setting.

The previous screening step revealed which phytohormones are part of a JA-dependent network regulating anther development in *L. luteus* under well-watered conditions. Then, we investigated these selected phytohormones in detail, measuring their levels under both well-watered and drought stress conditions. This final step further supports the involvement of these JA-interacting pathways in the context of drought. The phases in which stress conditions significantly increased SA and GA concentrations were LAD1 and LAD4. In the remaining LAD phases, drought did not induce such changes, and its impact was negligible or decreased the amount of phytohormone. However, IAA levels remained unchanged during anther dehiscence under drought conditions but decreased during senescence. Therefore, our results suggest that drought stress disturbs the balance of phytohormones interacting with JA during anther development in the tested species.

Differences in the morphological development of *L. luteus* stamens under various environmental conditions were noticeable, and may be related to phytohormonal disturbances. In other plant species, the unelongated filaments of stamens and indehiscent anthers are often due to reduced GA levels caused by mutations within genes encoding the GA biosynthetic enzymes (Song et al. 2013; Marciniak and Przedniczek 2019). Regarding *L. luteus*, we observed that drought stress reduced the GA_7_ level during anther dehiscence. Moreover, in both *A. thaliana* and *L. luteus*, GAs interact with JAs to regulate late stamen development (Song et al. 2013; Marciniak et al. 2024). On the morphological level of entire plant, drought caused significant shortening of the *L. luteus* stem and inhibited the development of leaves, roots, and inflorescence. In other species, the inhibition of stem elongation is associated with reduced GA levels under stress conditions (Litvin et al. 2016), which was also observed in *L. luteus*.

For a broader overview, we also assessed the effect of drought stress on the phytohormone levels in *L. luteus* flowers, leaves, stems, and roots. These studies showed significant differences depending on the tissue type (vegetative vs. reproductive), developmental stage, and environmental conditions. In flowers, JA concentrations were largely unaffected by drought, IAA levels were phase-specific, while the SA balance was disturbed and reversed. Although drought led to a moderate increase in GA levels, a noticeable decline in GA_3_ and GA_7_ contents were observed specifically during the F5 phase. Certainly, flowers developing under optimal conditions accumulated the highest GA levels in the F5 phase. This aligns with results from our previous work indicating that the transcriptional activity of *LlDELLA1*, which encodes a protein acting as the main repressor of GA signaling, is the lowest in the F5 phase, i.e., after anther dehiscence and during pollination/fertilization (Marciniak and Przedniczek 2020). This suggests that the correlation between *LlDELLA1* and GA_3/7_ may be important for proper flower and very early seed/pod development. In vegetative organs like leaves, drought increased the levels of JA, SA, GA_3_ (lower leaves) and GA_7_ (upper leaves). This could be related to the regulation of osmotic balance, stomata closure, or redox shift, as confirmed in other species (Iqbal et al. 2022). In *L. luteus* stems, drought decreased the concentrations of JA, SA, and GAs, which is likely the reason for a significant reduction in stem elongation/growth restriction in *L. luteus*, as well as other plants (Colebrook et al. 2014). Additionally, drought significantly declined the content of phytohormones (excluding SA) in *L. luteus* roots. It has also been shown that the level of transcriptional activity of JA biosynthesis and perception genes in flowers and vegetative organs developing under unfavorable environmental conditions is tightly correlated with a specific developmental phase.

In summary, our findings highlight the crucial role of phytohormonal balance in *L. luteus* anther development and plant adaptation to drought stress. Understanding the processes underlying pollen release at the hormonal, cytological, and molecular levels is an essential aspect in controlling fertility in economically important legume species and increased interest among breeders.

## Materials and methods

### Plant material, growth conditions, and compound treatments

As plant material, the restricted branching yellow lupin (*Lupinus luteus* L.) cv. Taper was used. Seeds were provided by the Poznańska Hodowla Roślin Ltd (Wiatrowo Branch, Poland), prepared following the protocol described by Marciniak et al. (2024), and sown at the turn of March and April into 10 dm^3^ pots containing fifth-class soil (Polish bonitation classification of soils) and a mineral NPK fertilizer for legumes (Polifoska 6, NPK 6–20–30 + 7 SO₃, Grupa Azoty, Police, Poland). The plants were cultivated outdoors (Pędzewo, Poland, 53° 05′ 02″ N 18° 21′ 28″ E) under a polytunnel, which allowed for control of soil moisture. Initially, optimal hydration conditions (100% of field capacity, FC) were maintained for 30 days. Then, the plants were divided into two experimental groups: a well-watered group, where hydration was constantly maintained at 100% FC, and a drought-stressed group, where soil moisture was reduced to 30% FC. The experiment utilized a modified Imakumbili et al. (2021) soil moisture adjustment method, specifically designed to impose water stress conditions. FC was established by saturating air-dried soil in perforated pots protected against evaporation and allowing excess water to drain under controlled conditions. The mass of the moist soil was recorded after 3 days and compared to its oven-dried mass to determine FC. This value served as a reference for calculating water volumes required to achieve specific moisture levels. Pots were weighed regularly, and water was added as needed to achieve the calculated FC. To ensure accuracy, control pots containing the same plants were also prepared. These control pots allowed for the estimation of plant weight, which was used to adjust water volume added to pots accounting for both the plant and pot weights, thereby ensuring precise maintenance of the desired FC.

Flowers (F1-2, F3-4, F5, F6-7, F8) and anthers (LAD1-5, Late Anther Development) from five developmental stages were dissected from no less than 90 randomly selected plants, and vegetative organs (upper leaves, lower leaves, stems, roots) were collected from no less than 30 randomly selected plants. Upper and lower leaves were sampled simultaneously from the apical and basal regions, respectively, of well-watered and drought-treated plants at the generative developmental stage. Samples were processed fresh or frozen in liquid nitrogen, depending on experiment. The part of the anthers being in the LAD3 phase was treated with the JA biosynthesis inhibitor—mefenamic acid (MEF, 2-[(2,3-Dimethylphenyl)amino]benzoic acid) (Merck, Darmstadt, Germany) (Wilmowicz et al. 2016) diluted with water containing 0.05% Tween 20 (Merck) to a final concentration of 100 µM. The solution was stirred on a magnetic stirrer for at least 1 h. The anthers used as a control were treated with a 0.05% Tween 20 solution only. After application of the compound (3 h), the anthers were harvested, frozen in liquid nitrogen, and stored at −80 until use.

### RNA sequencing, de novo transcriptome assembly, and bioinformatic analyses

Total RNA was isolated using the NucleoSpin RNA kit (Macherey–Nagel, Düren, Germany). Two isolations were performed from *L. luteus* anthers (LAD3 phase) collected from at least 90 randomly selected plants for each variant—treated with MEF or untreated. The concentration and purity were measured with the NanoDrop ND-1000 spectrophotometer (Thermo Fisher Scientific, Waltham, MA, USA) before further analysis. The integrity and quality of total RNA were assessed using the Agilent 2100 Bioanalyzer (Agilent Technologies, Santa Clara, CA, USA) and agarose gel electrophoresis. Samples with RNA integrity number (RIN) > 8 were selected for further procedures. The cDNA library construction and the transcriptome sequencing were carried out by Genomed (Warsaw, Poland) on the HiSeq 2000 platform (Illumina Inc., San Diego, CA, USA). The data have been deposited in the Sequence Read Archive (SRA) database at NCBI under the accession number PRJNA1201074 (BioProject). Quality of the raw reads was assessed using FastQC (ver 0.11.5), then separate quality filtering and adapter trimming were performed with BBDUK2 (ver 38.32) with the following parameters: qtrim = w, trimq = 20, maq = 10, *k* = 23, min*k* = 11, hdist = 1, tbo, tpe, minlength = 50. Acquired reads were mapped to *L. angustifolius* ribosomal RNA sequences (NCBI RefSeq GCF_001865875.1) and filtered out. The remaining reads were used for *de-novo* sequence assembly using Trinity ver 2.8.5 with default parameters and in silico read normalization enabled. Each isoform was assigned an ID number consisting of the cluster number (c), the number of genes within the given cluster (g), and the isoform number (i). For functional transcriptome annotation, Trinotate (ver. 3.0.2) was used following its standard workflow. First, transcript sequences were searched against the Swiss‑Prot database using BLASTX (BLAST +; max_target_seqs = 1). Open reading frames were predicted with TransDecoder (ver. 5.0.1), and the resulting protein sequences were queried against Swiss‑Prot using BLASTP (max_target_seqs = 1). Protein domains were identified with hmmscan (HMMER) using default parameters and the Pfam database. Annotation tables were then integrated within Trinotate, and UniProt-derived metadata, including Gene Ontology (GO) terms, were used to assign functional categories to transcripts. Expression values were obtained with Salmon (ver 0.9.1) using default settings except for additional bias corrections (–seqBias and –gcBias) aimed at providing improved performance. Differential expression analysis was performed using Ebseq (ver 1.26.0) with default settings, applying a posterior probability of equal expression (PPEE) < 0.05 together with an absolute value of fold change (|Log2FC|) > 2 as significance cutoffs (Leng et al. 2013). Gene set enrichment analysis (GSEA) was performed with goseq (ver 1.24.0) using default bias correction for gene length. Gogadget (ver 2.1) was then used to filter and rearrange redundant GO terms, as well as generate GO term network maps for further Cytoscape analyses. GO terms where visualized as Enrichment Maps with the Cytoscape with Enrichment Map addon.

### Liquid chromatography with tandem mass spectrometry

Liquid chromatography with tandem mass spectrometry (LC–MS/MS) was used to determine the concentrations of JA, GA_3_, GA_7_, SA, and IAA in reproductive and vegetative organs of *L. luteus* growing under different conditions. Phytohormones were extracted according to the previously described protocol (Marciniak et al. 2024). The following amounts of internal standards were used: 8 ng µl^−1^ d5JA (C/D/N Isotopes, Quebec, Canada), 5 ng µl^−1^ d2GA_3_, 5 ng µl^−1^ d2GA_7_ (OlChemIm, Olomouc, Czech Republic), 10 ng µl^−1^ d4SA, and 5 ng µl^−1^ d2IAA (Merck) were used. The analyses were carried out on a Shimadzu Nexera XR UHPLC system coupled with a Shimadzu LC–MS-8045 triple-quadrupole mass spectrometer (Shimadzu, Kyoto, Japan). Separation was performed on a Ascentis Express 90 Å C-18 column (2.7 μm, 100 × 2.1 mm, Supelco, Bellefonte, PA, USA). The m/z multiple reaction monitoring transitions were selected: 211.1/133.3 for JA, 214.2/134.3 for d5JA, 345.3/329.3 for GA_3_, 347.3/241.3 for d2GA_3_, 329.1/323.3 for GA_7_, 331.4/325.3 for d2GA_7_, 137.3/93.3 for SA, 141.4/97.1 for d4SA, 176.2/130.3 for IAA, and 178.2/132.3 for d2IAA. Data analyses were performed using LabSolutions software 5.8 (Shimadzu).

### Histological and immunohistochemical methods

Anthers being in LAD2 and LAD4 phases were dissected from *L. luteus* flowers growing in well-watered and drought stress conditions, fixed [solution of 4% paraformaldehyde (w/v), 0.25% glutaraldehyde (v/v), 2% N-ethyl-N′-(3-dimethylaminopropyl) carbodiimide hydrochloride (EDAC) (w/v) (Merck) in 1 × phosphate-buffered saline buffer (PBS, pH 7.2)], dehydrated in increasing ethanol concentrations with dithiothreitol, gradually supersaturated, and embedded in BMM resin in accordance with a previously established procedure (Marciniak et al. 2024). Samples were cut into 1.5 µm thick sections using an ultramicrotome (Leica, Wetzlar, Germany) and placed on silanized glass slides (Pathosolutions, Elektro Med, Poland). Histological staining was performed with 0.05% toluidine blue (Merck) and the preparations were observed using a Zeiss Axioplan Light Microscope (Carl Zeiss, Oberkochen, Germany) coupled to a ProGres C3 digital camera. Before IHC analyses, the sections were washed, BlockAid™ Blocking Solution (Thermo Fisher Scientific) was used, and the Image-iT FX Signal Enhancer (Cell Signaling Technology, Danvers, MA, USA) was applied, according to the instructions developed in our previous research (Marciniak et al. 2024). Anti-JA primary antibody (product no AS11 1799, Agrisera, Vännäs, Sweden) and secondary antibody (MFP488 goat anti-rabbit IgG, product no MFP-A1008, MoBiTec, Goettingen, Germany), both diluted in BlockAid Blocking Solution, were used. Then, incubation with DAPI was performed, and ProLong Diamond Antifade Mountant (Thermo Fisher Scientific) was applied to the slides. The samples were observed under a Leica DMI4000B inverted microscope using BP 365, FT 395 and LP 397 filters. A negative control was performed by omitting incubation with the primary antibody (Fig. S9).

### RNA isolation, reverse transcription, qPCR, and validation of RNA-Seq results

Total RNA was isolated from 80–100 mg of reproductive and vegetative organs of *L. luteus* growing under well-watered and drought stress conditions, with or without MEF treatment, by the column method with the use of (1) RNeasy Plant Mini Kit (Qiagen, Germantown, MD, USA) (expression of JA biosynthesis and perception genes, Fig. 4) or (2) NucleoSpin RNA kit (Macherey–Nagel) (RNA-Seq validation, Fig. S10). In both cases, for the reverse transcription (RT) reactions, 2 μg of total RNA as template, anchored-oligo(dT)_18_ primers, and the Transcriptor First Strand cDNA Synthesis Kit (Roche, Mannheim, Germany) were used, adhering to the manufacturer’s guidelines. All qPCRs and analyses were performed according to the method described by Marciniak and Przedniczek 2021 and Marciniak et al. 2024. The following accession numbers (GenBank, NCBI) have been assigned to individual sequences: OQ989994 (*LlAOS*), OQ989993 (*LlAOC*), OQ989987 (*LlOPR3*), and OQ989980 (*LlCOI1*), which cDNAs were obtained from RNA-Seq experiment deposited at the NCBI in the SRA database under accession number PRJNA285604 (BioProject) and experiment accession number SRX1069734. The Universal Probe Library Assay Design Center and Primer3 software were used to design specific primers (Tables S1−S2) and matching Universal Probe Library (UPL) hydrolysis probes (Tables S1−S2). Two kits were used in the reactions depending on whether the UPL probe or the SYBR Green dye was used as a fluorescence detection system: LightCycler TaqMan Master or LightCycler FastStart DNA Master SYBR Green I (Roche). Each reaction contained: 0.1 μg of cDNA, 0.2 μM gene specific primers, 0.05 μM UPL probe, and 1 × LightCycler TaqMan Master Mix; or 0.1 μg of cDNA, 0.2 μM gene-specific primers, 4 mM Mg^2+^, and 1 × LightCycler FastStart DNA Master SYBR Green I Mix. Distilled water was added up to 20 μl in both cases. The reactions were carried out according to the programs presented in the tables S1−S2 with the use of LightCycler 2.0 Carousel-Based System connected with LightCycler Software (Roche). The actin gene (*LlACT*, acc. no KP257588) was chosen as endogenous control (Glazińska et al. 2017; Marciniak et al. 2018a, b, c; Marciniak and Przedniczek 2020, 2021; Marciniak et al. 2024). Statistical analysis was performed using Welch’s ANOVA followed by the Games-Howell post hoc test. Significance was assessed at a threshold of *p* < 0.05, indicating statistically significant differences. Statistical analysis was conducted using the “rstatix” package in R. The data are presented as the mean ± SD and consist of three biological replicates with two technical replicates.

## Supplementary Information

Below is the link to the electronic supplementary material.

Supplementary file1 (PDF 1642 KB)

## Data Availability

The data that supports the findings of this study are available in the supplementary material of this article. The transcriptomic data have been deposited in the SRA database at NCBI under the accession number PRJNA1201074 (BioProject). The following accession numbers (GenBank, NCBI) have been assigned to individual sequences: OQ989994 (*LlAOS*), OQ989993 (*LlAOC*), OQ989987 (*LlOPR3*), and OQ989980 (*LlCOI1*), which cDNAs were obtained from RNA-Seq experiment deposited at the NCBI in the SRA database under accession number PRJNA285604 (BioProject) and experiment accession number SRX1069734.
