# Supplementary material for: Drought alters jasmonate-dependent regulation of anther development in yellow lupin (Lupinus luteus L.)
Source: Planta. 2026 Aug 1;264(3):79. doi: 10.1007/s00425-026-05107-7 (PMC13428782; doi:10.1007/s00425-026-05107-7)
Supplement: Supplementary file 1 — Supplementary file1 (PDF 1642 KB) [file 425_2026_5107_MOESM1_ESM.pdf]

MEF-CONTROL, **CARBOHYDRATE METABOLISM**

A

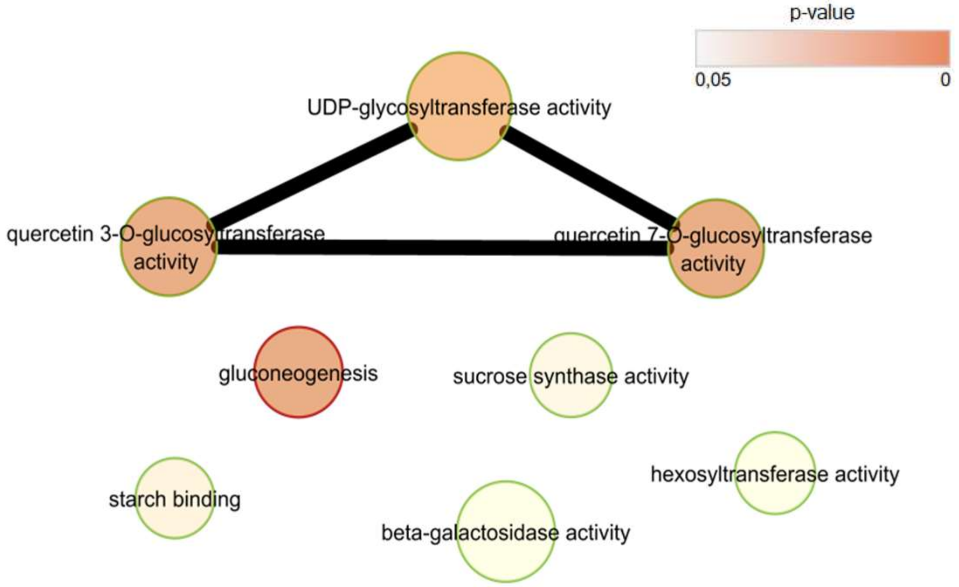

B

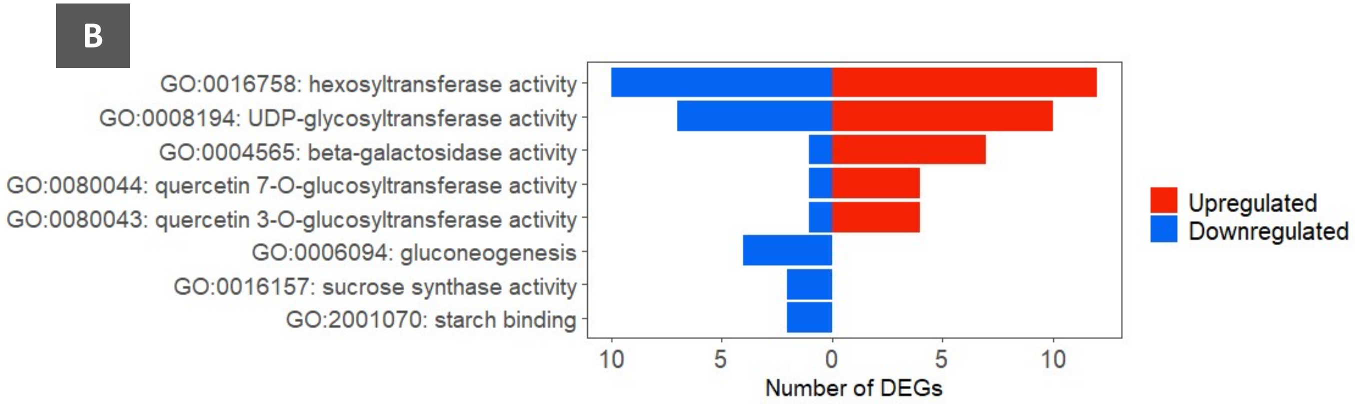

Fig. S1. Changes in the expression profile of genes associated with the carbohydrate metabolism in MEF-Control comparison.

(A) The gene enrichment map obtained using the EnrichmentMap tool for Cytoscape. Nodes represent groups of genes with a common biological meaning described by GO terms. The node size is proportional to the number of genes with a given biological meaning in the transcriptome. The node color reflects the statistical significance level (p-value) for GO term enrichment, and the node outline color indicates the GO term category for a given node, where green represents "molecular function" term and red represents "biological process" term. Edges connect nodes that share a portion of genes. The edge thickness is proportional to the number of shared genes between nodes for the entire transcriptome;

(B) A bar chart showing the number of differentially expressed genes (up- and downregulated) annotated with the analyzed GO terms;

(C) The annotations of various unigenes obtained from Swissprot data using the BlastP and BlastX programs.

MEF-CONTROL, **CARBOHYDRATE METABOLISM**

C

Gene Id	No. of Unigenes	GO Annotations	Annotation Id	Blast Annotation Full Name	$Log_{10}(FC)$
TRINITY_DN15731_c4_g7	1	GO:0008194, GO:0016758	HQGT_RAUSE	Hydroquinone glucosyltransferase	1,42037951
TRINITY_DN18237_c3_g1	6	GO:0004565	BGAL1_ARATH<br>BGAL_SOLLC	Beta-galactosidase 1<br>Beta-galactosidase	1,164210101
TRINITY_DN37112_c0_g1	1	GO:0016758	XTH2_ARATH	Xyloglucan endotransglucosylase/hydrolase protein 2	1,15640116
TRINITY_DN10589_c1_g7	1	GO:0008194, GO:0016758	GATL1_ARATH	Probable galacturonosyltransferase-like 1	1,122415502
TRINITY_DN13321_c0_g4	3	GO:0008194, GO:0016758	BC10_ORYSJ	Glycosyltransferase BC10 {ECO:0000305}	1,122243087
TRINITY_DN7993_c0_g1	4	GO:0004565	BGAL8_ARATH	Beta-galactosidase 8	1,100926452
TRINITY_DN11551_c2_g3	1	GO:0008194, GO:0016758	UGT2_GARJA	7-deoxyloganetin glucosyltransferase	1,092562052
TRINITY_DN13988_c4_g1	8	GO:0004565	BGA16_ARATH<br>BGAL6_ARATH	Beta-galactosidase 16<br>Beta-galactosidase 6	1,076673357
TRINITY_DN4379_c0_g2	1	GO:0016758	XTH26_ARATH	Probable xyloglucan endotransglucosylase/hydrolase protein 26	1,066685863
TRINITY_DN17342_c0_g1	1	GO:0004565	BGAL_ASPOF	Beta-galactosidase	1,0133135
TRINITY_DN12196_c1_g1	2	GO:0008194, GO:0016758,<br>GO:0080043, GO:0080044	U83A1_ARATH	UDP-glycosyltransferase 83A1	0,903475942
TRINITY_DN10991_c4_g6	3	GO:0008194, GO:0016758	HQGT_RAUSE	Hydroquinone glucosyltransferase	0,870759056
TRINITY_DN14918_c1_g1	6	GO:0004565	BGAL6_ARATH	Beta-galactosidase 6	0,794946123
TRINITY_DN7760_c0_g1	4	GO:0008194, GO:0016758	UFOG5_MANES	Anthocyanidin 3-O-glucosyltransferase 5	0,794121558
TRINITY_DN7548_c0_g1	2	GO:0008194, GO:0016758,<br>GO:0080043, GO:0080044	U83A1_ARATH	UDP-glycosyltransferase 83A1	0,792196813
TRINITY_DN18237_c3_g2	23	GO:0004565	BGAL1_ARATH<br>BGAL_SOLLC	Beta-galactosidase 1<br>Beta-galactosidase	0,788170221
TRINITY_DN16440_c0_g1	1	GO:0008194, GO:0016758,<br>GO:0080043, GO:0080044	U74E1_ARATH	UDP-glycosyltransferase 74E1	0,783297543
TRINITY_DN14775_c2_g1	2	GO:0008194, GO:0016758,<br>GO:0080043, GO:0080044	U74D1_ARATH<br>U74E2_ARATH	UDP-glycosyltransferase 74D1<br>UDP-glycosyltransferase 74E2	0,66860636
TRINITY_DN13166_c2_g1	7	GO:0004565	BGAL1_ARATH<br>BGAL4_ARATH	Beta-galactosidase 1<br>Beta-galactosidase 4	0,663602148

RAUSE - *Rauvolfia serpentina,* ARATH - *Arabidopsis thaliana*, SOLLC - *Solanum lycopersicum*, ORYSJ - *Oryza sativa*, GARJA - *Gardenia jasminoides*, ASPOF - *Asparagus officinalis*, MANES - *Manihot esculenta*

MEF-CONTROL, **CARBOHYDRATE METABOLISM**

C

Gene Id	No. of Unigenes	GO Annotations	Annotation Id	Blast Annotation Full Name	Log_{10}(FC)
TRINITY_DN15024_c0_g2	32	GO:0006094	PCKA1_UROPA<br>PCKA_ARATH<br>PCKA_CUCSA	Phosphoenolpyruvate carboxykinase (ATP) 1<br>Phosphoenolpyruvate carboxykinase (ATP)<br>Phosphoenolpyruvate carboxykinase (ATP)	-0,58611602
TRINITY_DN12204_c0_g1	6	GO:0006094	PCKA2_UROPA<br>PCKA_ARATH<br>PCKA_MAIZE	Phosphoenolpyruvate carboxykinase (ATP) 2<br>Phosphoenolpyruvate carboxykinase (ATP)<br>Phosphoenolpyruvate carboxykinase (ATP)	-0,58888252
TRINITY_DN18063_c1_g1	2	GO:0008194, GO:0016157, GO:0016758	SUS2_PEA<br>SUS3_ARATH	Sucrose synthase 2<br>Sucrose synthase 3	-0,64941113
TRINITY_DN15120_c0_g1	7	GO:0008194, GO:0016157, GO:0016758	SUS2_PEA	Sucrose synthase 2	-0,65808271
TRINITY_DN17110_c1_g5	3	GO:0008194, GO:0016758, GO:0080043, GO:0080044	FG3H_SOYBN<br>FG3N_SOYBN	UDP-glycosyltransferase 79B30 {ECO:0000303|PubMed:26002063}<br>UDP-glycosyltransferase 79B30 {ECO:0000303|PubMed:26002063}	-0,69805923
TRINITY_DN15852_c1_g4	1	GO:0016758	FUT1_ARATH	Galactoside 2-alpha-L-fucosyltransferase	-0,70535334
TRINITY_DN12051_c1_g1	3	GO:0008194, GO:0016758	CESA1_ARATH	Cellulose synthase A catalytic subunit 1 [UDP-forming]	-0,77639845
TRINITY_DN12058_c2_g9	3	GO:2001070	AMYG_ASPOR	Glucoamylase	-0,79515153
TRINITY_DN12204_c0_g3	1	GO:0006094	PCKA_ARATH	Phosphoenolpyruvate carboxykinase (ATP)	-0,88644068
TRINITY_DN8671_c0_g2	1	GO:0016758	FUT1_ARATH	Galactoside 2-alpha-L-fucosyltransferase	-0,91049167
TRINITY_DN17815_c3_g2	1	GO:0008194, GO:0016758	CESA1_ORYSJ	Probable cellulose synthase A catalytic subunit 1 [UDP-forming]	-0,92955197
TRINITY_DN11139_c7_g1	1	GO:0008194, GO:0016758	BC10_ORYSJ	Glycosyltransferase BC10 {ECO:0000305}	-0,93677867
TRINITY_DN11545_c2_g1	1	GO:0008194, GO:0016758, GO:2001070	SSY3_SOLTU	Soluble starch synthase 3, chloroplastic/amyloplastic	-0,98158441
TRINITY_DN15024_c0_g4	1	GO:0006094	PCKA_ARATH	Phosphoenolpyruvate carboxykinase (ATP)	-1,10245819
TRINITY_DN15999_c1_g2	5	GO:0016758	AA5GT_DIACA<br>BGL11_ARATH	Cyanidin 3-O-glucoside 5-O-glucosyltransferase (acyl-glucose)<br>Beta-glucosidase 11	-1,11518497
TRINITY_DN17107_c1_g3	3	GO:0004565	BGA11_ARATH<br>BGA14_ARATH	Beta-galactosidase 11<br>Beta-galactosidase 14	-1,12819062

UROPA - *Urochloa panicoides*, ARATH - *Arabidopsis thaliana*, CUCSA - *Cucumis sativus,* MAIZE - *Zea mays*, PEA - *Pisum sativum*, SOYBN - *Glycine max*, ASPOR - *Aspergillus oryzae*, ORYSJ - *Oryza sativa*, SOLTU - *Solanum tuberosum*, DIACA - *Dianthus caryophyllus*

MEF-CONTROL, **CHITINASES and PECTINESTERASES**, i.e. enzymes related to the breakdown of carbohydrates

A

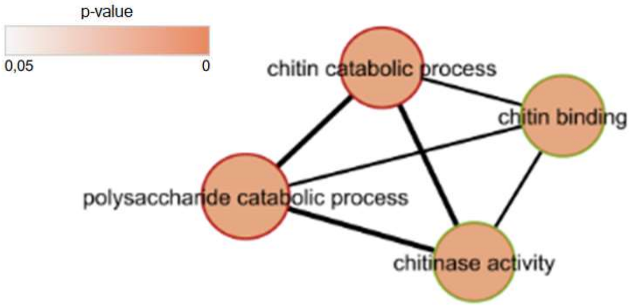

B

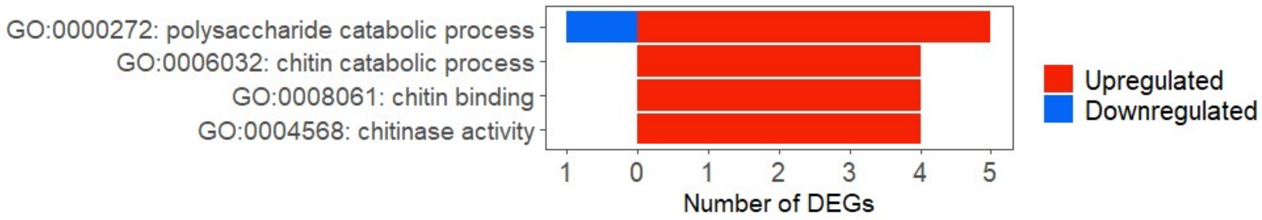

C

Gene Id	No. of unigenes	GO Annotations	Annotation Id	Blast Annotation Full Name	$Log_{10}(FC)$
TRINITY_DN5196_c0_g1	1	GO:0000272, GO:0004568, GO:0006032	CHIA_TOBAC	Acidic endochitinase	1,627260346
TRINITY_DN15184_c2_g1	11	GO:0000272	PME10_ARATH<br>PME29_ARATH<br>PME52_ARATH<br>PME66_ARATH	Putative pectinesterase 10<br>Probable pectinesterase 29<br>Putative pectinesterase 52<br>Probable pectinesterase 66	1,283091172
TRINITY_DN11852_c4_g5	3	GO:0000272, GO:0004568, GO:0006032, GO:0008061	CHIC_ARATH	Class V chitinase {ECO:0000303|PubMed:22936594}	1,137510386
TRINITY_DN12299_c7_g1	3	GO:0008061	CHIT5_LOTJA	Class V chitinase CHIT5 {ECO:0000303|PubMed:27383628}	1,087644451
TRINITY_DN8059_c0_g1	2	GO:0000272, GO:0004568, GO:0006032	CHIA_CICAR	Acidic endochitinase	0,955803577
TRINITY_DN23283_c0_g1	1	GO:0000272, GO:0004568, GO:0006032, GO:0008061	CHIC_ARATH	Class V chitinase {ECO:0000303|PubMed:22936594}	0,84218033
TRINITY_DN12858_c0_g4	2	GO:0008061	HEVE_HEVBR	Pro-hevein	0,740093934
TRINITY_DN12058_c2_g9	3	GO:0000272	AMYG_ASPOR	Glucoamylase	-0,79515153

TOBAC - *Nicotiana tabacum*
ARATH - *Arabidopsis thaliana*
LOTJA - *Lotus japonicus*
CICAR - *Cicer arietinum*
HEVBR - *Hevea brasiliensis*
ASPOR - *Aspergillus oryzae*

Fig. S2. Changes in the expression profile of genes encoded enzymes related to the breakdown of carbohydrates in MEF-Control comparison.
(A) The gene enrichment map obtained using the EnrichmentMap tool for Cytoscape. Nodes represent groups of genes with a common biological meaning described by GO terms. The node size is proportional to the number of genes with a given biological meaning in the transcriptome. The node color reflects the statistical significance level (p-value) for GO term enrichment, and the node outline color indicates the GO term category for a given node, where green represents “molecular function” term and red represents “biological process” term. Edges connect nodes that share a portion of genes. The edge thickness is proportional to the number of shared genes between nodes for the entire transcriptome;
(B) A bar chart showing the number of differentially expressed genes (up- and downregulated) annotated with the analyzed GO terms;
(C) The annotations of various unigenes obtained from Swissprot data using the BlastP and BlastX programs.

MEF-CONTROL, CELL WALL ORGANIZATION

A

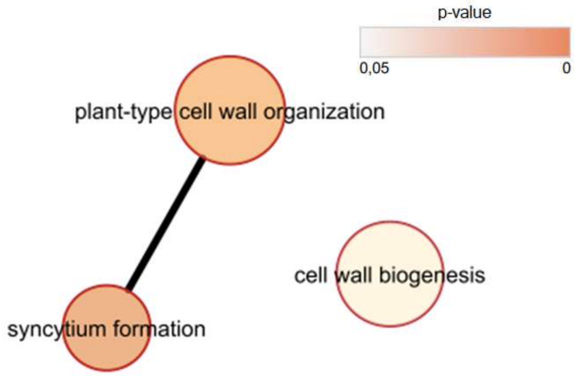

B

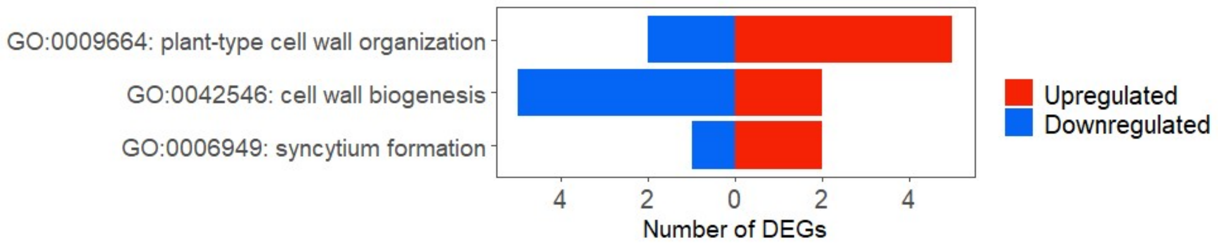

C

Gene Id	No. of unigenes	GO Annotations	Annotation Id	Blast Annotation Full Name	Log_{10}(FC)
TRINITY_DN37112_c0_g1	1	GO:0042546	XTH2_ARATH	Xyloglucan endotransglucosylase/hydrolase protein 2	1,15640116
TRINITY_DN12245_c2_g4	2	GO:0009664	MYB87_ARATH	Transcription factor MYB87 {ECO:0000305}	1,132663622
TRINITY_DN12565_c3_g2	1	GO:0006949, GO:0009664	EXPA4_ARATH	Expansin-A4	1,111765259
TRINITY_DN13988_c4_g1	8	GO:0009664	BGA16_ARATH<br>BGAL6_ARATH	Beta-galactosidase 16<br>Beta-galactosidase 6	1,076673357
TRINITY_DN4379_c0_g2	1	GO:0042546	XTH26_ARATH	Probable xyloglucan endotransglucosylase/hydrolase protein 26	1,066685863
TRINITY_DN13805_c5_g1	1	GO:0006949, GO:0009664	EXPA4_ARATH	Expansin-A4	0,931958783
TRINITY_DN14918_c1_g1	6	GO:0009664	BGAL6_ARATH	Beta-galactosidase 6	0,794946123
TRINITY_DN15852_c1_g4	1	GO:0042546	FUT1_ARATH	Galactoside 2-alpha-L-fucosyltransferase	-0,70535334
TRINITY_DN11607_c3_g2	2	GO:0006949, GO:0009664	EXP14_ARATH<br>EXP16_ARATH	Expansin-A14<br>Expansin-A16	-0,7234389
TRINITY_DN12051_c1_g1	3	GO:0042546	CESA1_ARATH	Cellulose synthase A catalytic subunit 1 [UDP-forming]	-0,77639845
TRINITY_DN8671_c0_g2	1	GO:0042546	FUT1_ARATH	Galactoside 2-alpha-L-fucosyltransferase	-0,91049167
TRINITY_DN17815_c3_g2	1	GO:0042546	CESA1_ORYSJ	Probable cellulose synthase A catalytic subunit 1 [UDP-forming]	-0,92955197
TRINITY_DN7991_c0_g1	3	GO:0009664, GO:0042546	COBL3_ORYSJ<br>COBL6_ARATH	COBRA-like protein 3<br>COBRA-like protein 6	-1,05302951

ARATH - *Arabidopsis thaliana,* ORYSJ - *Oryza sativa*

Fig. S3. Changes in the expression profile of genes associated with the cell wall modification in MEF-Control comparison. **(A)** The gene enrichment map obtained using the EnrichmentMap tool for Cytoscape. Nodes represent groups of genes with a common biological meaning described by GO terms. The node size is proportional to the number of genes with a given biological meaning in the transcriptome. The node color reflects the statistical significance level (p-value) for GO term enrichment, and the node outline red color indicates the GO term category ("biological process"). An edge connects nodes that share a portion of genes. The edge thickness is proportional to the number of shared genes between nodes for the entire transcriptome; **(B)** A bar chart showing the number of differentially expressed genes (up- and downregulated) annotated with the analyzed GO terms; **(C)** The annotations of various unigenes obtained from Swissprot data using the BlastP and BlastX programs.

MEF-CONTROL, **LIPID METABOLISM AND TRANSPORT**

A

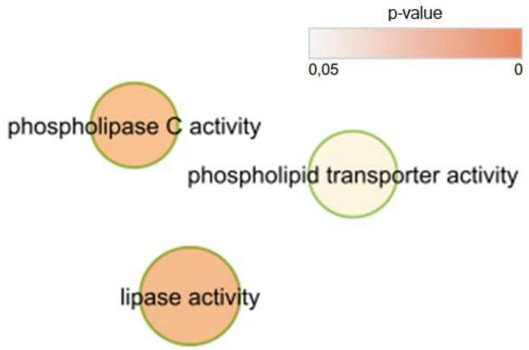

B

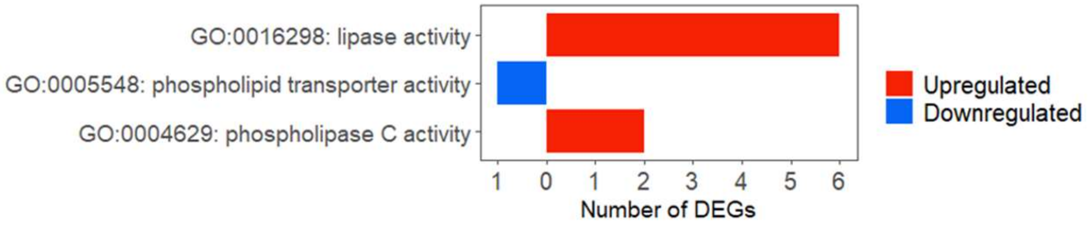

C

Gene Id	No. of unigenes	GO Annotations	Annotation Id	Blast Annotation Full Name	Log_{10}(FC)
TRINITY_DN14340_c4_g4	6	GO:0016298	EXL2_ARATH<br>EXL3_ARATH	GDSL esterase/lipase EXL2<br>GDSL esterase/lipase EXL3	1,022740595
TRINITY_DN12248_c2_g1	8	GO:0016298	GDL87_ARATH	GDSL esterase/lipase At5g55050	0,803112446
TRINITY_DN15745_c6_g3	3	GO:0004629, GO:0016298	NPC3_ARATH<br>NPC4_ARATH	Non-specific phospholipase C3<br>Non-specific phospholipase C4	0,778303529
TRINITY_DN13804_c0_g1	6	GO:0016298	EXL3_ARATH<br>GDL90_ARATH	GDSL esterase/lipase EXL3<br>GDSL esterase/lipase At5g42170	0,738714502
TRINITY_DN13067_c4_g1	3	GO:0016298	MES2_ARATH<br>SABP2_TOBAC	Methylesterase 2<br>Salicylic acid-binding protein 2	0,72493322
TRINITY_DN44489_c0_g1	1	GO:0004629, GO:0016298	NPC4_ARATH	Non-specific phospholipase C4	0,651485865
TRINITY_DN11218_c3_g4	1	GO:0005548	ALA3_ARATH	Phospholipid-transporting ATPase 3 {ECO:0000303|PubMed:11402198}	-1,10503334

ARATH - *Arabidopsis thaliana,* TOBAC - *Nicotiana tabacum*

Fig. S4. Changes in the expression profile of genes associated with the lipid metabolism in MEF-Control comparison.

(A) The gene enrichment map obtained using the EnrichmentMap tool for Cytoscape. Nodes represent groups of genes with a common biological meaning described by GO terms. The node size is proportional to the number of genes with a given biological meaning in the transcriptome. The node color reflects the statistical significance level (p-value) for GO term enrichment, and the node outline green color indicates the GO term category (“molecular function”);

(B) A bar chart showing the number of differentially expressed genes (up- and downregulated) annotated with the analyzed GO terms;

(C) The annotations of various unigenes obtained from Swissprot data using the BlastP and BlastX programs.

MEF-CONTROL, **TRANSPORT** – general overview

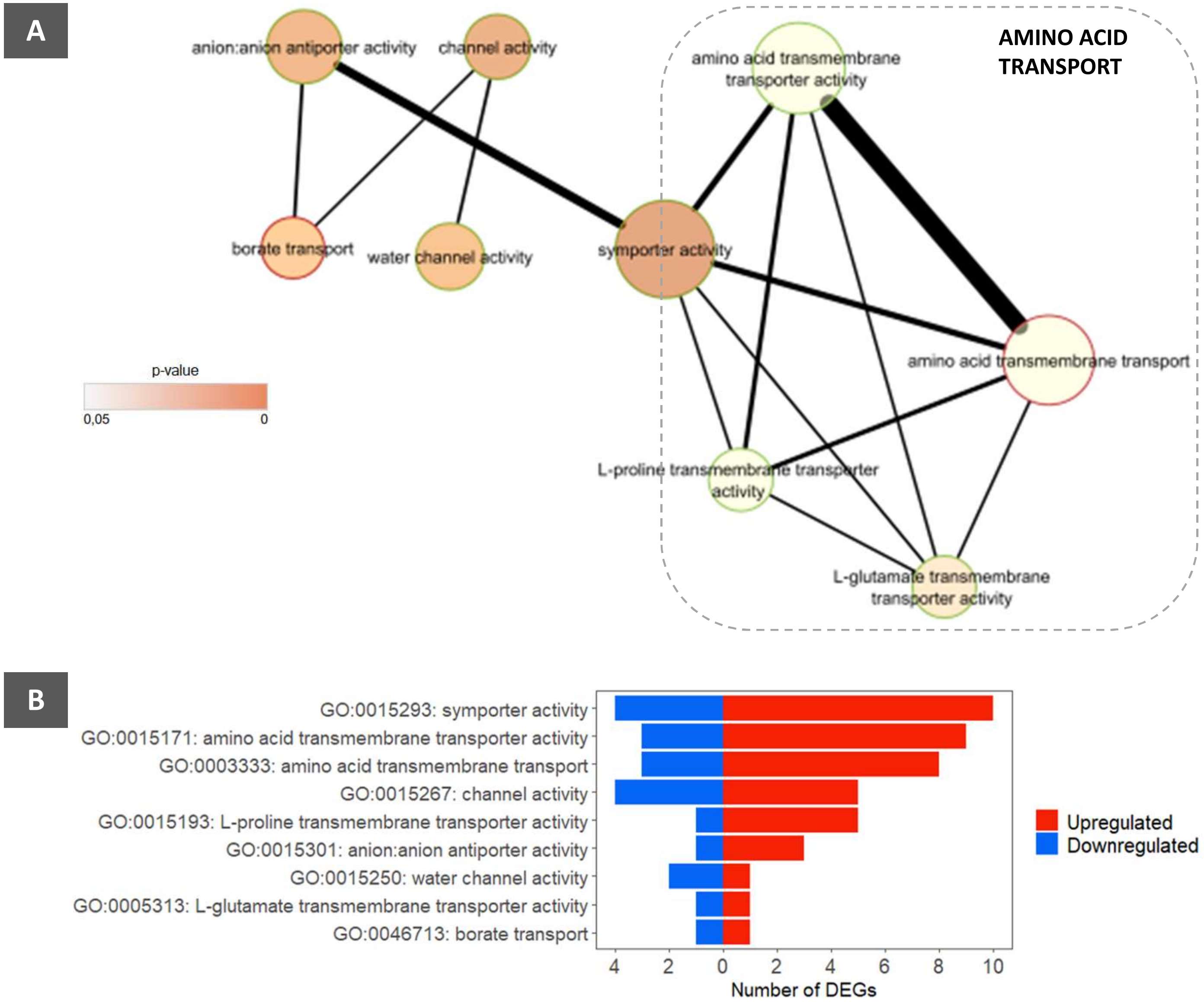

Fig. S5. Changes in the expression profile of genes associated with the transport of different substances in MEF-Control comparison.

(A) The gene enrichment map obtained using the EnrichmentMap tool for Cytoscape. Nodes represent groups of genes with a common biological meaning described by GO terms. The node size is proportional to the number of genes with a given biological meaning in the transcriptome. The node color reflects the statistical significance level (p-value) for GO term enrichment, and the node outline color indicates the GO term category for a given node, where green represents "molecular function" terms and red represents "biological process" terms. Edges connect nodes that share a portion of genes. The edge thickness is proportional to the number of shared genes between nodes for the entire transcriptome;

(B) A bar chart showing the number of differentially expressed genes (up- and downregulated) annotated with the analyzed GO terms;

(C) The annotations of various unigenes obtained from Swissprot data using the BlastP and BlastX programs.

MEF-CONTROL, **AMINO ACID TRANSPORT**

C1

Gene Id	No. of unigenes	GO Annotations	Annotation Id	Blast Annotation Full Name	$Log_{10}(FC)$
TRINITY_DN11509_c3_g2	8	GO:0003333, GO:0005313, GO:0015171, GO:0015193	AAP1_ARATH<br>AAP4_ARATH<br>AAP7_ARATH	Amino acid permease 1<br>Amino acid permease 4<br>Probable amino acid permease 7	0,922184406
TRINITY_DN16440_c1_g1	8	GO:0015171	CTNS_ARATH	Cystinosin homolog	0,745622657
TRINITY_DN15997_c3_g4	1	GO:0003333, GO:0015171, GO:0015193	PROT1_ARATH	Proline transporter 1	0,739497807
TRINITY_DN15997_c3_g3	2	GO:0003333, GO:0015171, GO:0015193	PROT2_ARATH	Proline transporter 2	0,727180797
TRINITY_DN13417_c1_g3	2	GO:0003333, GO:0015171, GO:0015193	PROT1_ARATH<br>PROT2_ARATH	Proline transporter 1<br>Proline transporter 2	0,722421366
TRINITY_DN13417_c1_g4	2	GO:0003333, GO:0015171, GO:0015193	PROT1_ARATH	Proline transporter 1	0,699477423
TRINITY_DN16410_c3_g2	8	GO:0003333, GO:0015171	AAP2_ARATH<br>AAP3_ARATH	Amino acid permease 2<br>Amino acid permease 3	0,681385075
TRINITY_DN18041_c0_g1	17	GO:0003333, GO:0015171	LHTL7_ARATH<br>LHTL8_ARATH	Lysine histidine transporter-like 7<br>Lysine histidine transporter-like 8	0,609722417
TRINITY_DN13233_c2_g2	3	GO:0003333, GO:0015171	AVT6A_ARATH	Amino acid transporter AVT6A {ECO:0000305}	0,605223833
TRINITY_DN14222_c0_g1	2	GO:0003333, GO:0015171	AAP6_ARATH<br>AAP8_ARATH	Amino acid permease 6<br>Amino acid permease 8	-0,67606793
TRINITY_DN11438_c2_g4	7	GO:0003333, GO:0005313, GO:0015171, GO:0015193	AAP1_ARATH<br>AAP6_ARATH<br>AAP8_ARATH	Amino acid permease 1<br>Amino acid permease 6<br>Amino acid permease 8	-0,71275331
TRINITY_DN7832_c0_g1	2	GO:0003333, GO:0015171	LHT1_ARATH<br>LHT2_ARATH	Lysine histidine transporter 1<br>Lysine histidine transporter 2	-0,87239361

MEF-CONTROL, **TRANSPORT – SYMPORTER ACTIVITY**

C2

Gene Id	No. of Unigenes	GO Annotations	Annotation Id	Blast Annotation Full Name	$Log_{10}(FC)$
TRINITY_DN12663_c0_g3	1	GO:0015293	SUT33_ARATH	Probable sulfate transporter 3.3	1,528370533
TRINITY_DN11826_c3_g3	2	GO:0015293	PTR19_ARATH	Protein NRT1/ PTR FAMILY 4.6	1,306848141
TRINITY_DN11024_c5_g2	1	GO:0015293	PLT2_ARATH<br>PLT5_ARATH	Putative polyol transporter 2<br>Polyol transporter 5	1,0319674
TRINITY_DN15483_c0_g2	6	GO:0015293	PTR1_ARATH<br>PTR5_ARATH	Protein NRT1/ PTR FAMILY 8.1<br>Protein NRT1/ PTR FAMILY 8.2	1,012497016
TRINITY_DN11509_c3_g2	8	GO:0015293	AAP1_ARATH<br>AAP4_ARATH<br>AAP7_ARATH	Amino acid permease 1<br>Amino acid permease 4<br>Probable amino acid permease 7	0,922184406
TRINITY_DN12663_c0_g1	5	GO:0015293	SUT33_ARATH	Probable sulfate transporter 3.3	0,78593296
TRINITY_DN11806_c2_g1	3	GO:0015293	SUT33_ARATH	Probable sulfate transporter 3.3	0,691989
TRINITY_DN16410_c3_g2	8	GO:0015293	AAP2_ARATH<br>AAP3_ARATH	Amino acid permease 2<br>Amino acid permease 3	0,681385075
TRINITY_DN11894_c5_g1	7	GO:0015293	PTR6_ARATH	Protein NRT1/ PTR FAMILY 1.2	0,66085377
TRINITY_DN13030_c1_g2	5	GO:0015293	PHT17_ARATH	Probable inorganic phosphate transporter 1-7	0,653194674
TRINITY_DN14222_c0_g1	2	GO:0015293	AAP6_ARATH<br>AAP8_ARATH	Amino acid permease 6<br>Amino acid permease 8	-0,67606793
TRINITY_DN11438_c2_g4	7	GO:0015293	AAP1_ARATH<br>AAP6_ARATH<br>AAP8_ARATH	Amino acid permease 1<br>Amino acid permease 6<br>Amino acid permease 8	-0,71275331
TRINITY_DN7832_c0_g1	2	GO:0015293	LHT1_ARATH<br>LHT2_ARATH	Lysine histidine transporter 1<br>Lysine histidine transporter 2	-0,87239361
TRINITY_DN12139_c0_g12	1	GO:0015293	PTR53_ARATH	Protein NRT1/ PTR FAMILY 2.11	-1,58260596

MEF-CONTROL, **TRANSPORT – ANTIPORTER ACTIVITY**

C3

Gene Id	No. of Unigenes	GO Annotations	Annotation Id	Blast Annotation Full Name	$Log_{10}(FC)$
TRINITY_DN12663_c0_g3	1	GO:0015301	SUT33_ARATH	Probable sulfate transporter 3.3	1,528370533
TRINITY_DN12663_c0_g1	5	GO:0015301	SUT33_ARATH	Probable sulfate transporter 3.3	0,78593296
TRINITY_DN11806_c2_g1	3	GO:0015301	SUT33_ARATH	Probable sulfate transporter 3.3	0,691989
TRINITY_DN14061_c0_g1	8	GO:0015301	BOR4_ARATH<br>BOR7_ARATH	Boron transporter 4<br>Probable boron transporter 7	-0,81141244

MEF-CONTROL, **TRANSPORT – CHANNEL ACTIVITY,** including water channel activity

C4

Gene Id	No. of Unigenes	GO Annotations	Annotation Id	Blast Annotation Full Name	Log_{10}(FC)
TRINITY_DN14503_c4_g1	5	GO:0015267	ALMT3_ARATH<br>ALMT9_ARATH	Putative aluminum-activated malate transporter 3<br>Aluminum-activated malate transporter 9	1,478381272
TRINITY_DN11934_c1_g1	10	GO:0015267	KAT1_ARATH<br>KAT2_ARATH	Potassium channel KAT1<br>Potassium channel KAT2	0,737637812
TRINITY_DN11236_c1_g1	15	GO:0015267	CNG20_ARATH<br>PGLR_VITVI<br>PRF1_SOLLC	Probable cyclic nucleotide-gated ion channel 20, chloroplastic<br>Probable polygalacturonase<br>36.4 kDa proline-rich protein	0,686945016
TRINITY_DN17733_c1_g2	6	GO:0015267	AMT23_ORYSJ<br>AMT2_ARATH<br>AMT31_ORYSJ	Ammonium transporter 2 member 3<br>Ammonium transporter 2<br>Ammonium transporter 3 member 1	0,67311627
TRINITY_DN11681_c0_g7	4	GO:0015250, GO:0015267, GO:0046713	NIP51_ARATH	Probable aquaporin NIP5-1	0,619224879
TRINITY_DN16093_c1_g1	1	GO:0015250, GO:0015267	TIP13_ARATH	Aquaporin TIP1-3	-0,63818524
TRINITY_DN15546_c2_g3	2	GO:0015267	NIP41_ARATH	Putative aquaporin NIP4-1	-0,7603077
TRINITY_DN7657_c0_g1	2	GO:0015250, GO:0015267	TIP13_ARATH	Aquaporin TIP1-3	-0,80654941
TRINITY_DN14061_c0_g1	8	GO:0046713	BOR4_ARATH<br>BOR7_ARATH	Boron transporter 4<br>Probable boron transporter 7	-0,81141244
TRINITY_DN15244_c0_g3	2	GO:0015267	ACA1_ARATH	Calcium-transporting ATPase 1	-0,82819113

ARATH - *Arabidopsis thaliana*, VITVI - *Vitis vinifera*, SOLLC - *Solanum lycopersicum*, ORYSJ - *Oryza sativa*

MEF-CONTROL, **BORATE TRANSPORT**

C5

Gene Id	No. of Unigenes	GO Annotations	Annotation Id	Blast Annotation Full Name	Log_{10}(FC)
TRINITY_DN11681_c0_g7	4	GO:0046713	NIP51_ARATH	Probable aquaporin NIP5-1	0,619224879
TRINITY_DN14061_c0_g1	8	GO:0046713	BOR4_ARATH<br>BOR7_ARATH	Boron transporter 4<br>Probable boron transporter 7	-0,81141244

MEF-CONTROL, **PROTEIN FOLDING**

A

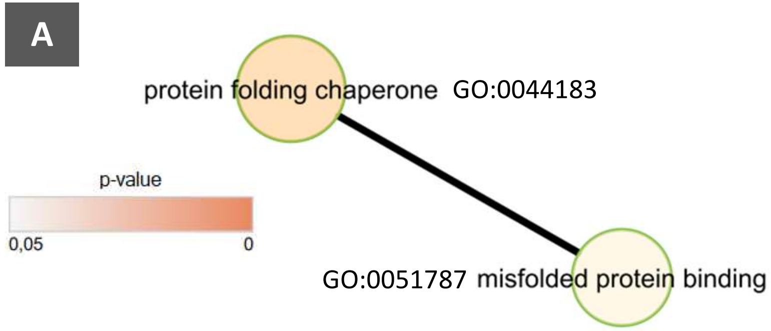

Fig. S6. Changes in the expression profile of genes associated with the protein folding in MEF-Control comparison.

(A) The gene enrichment map obtained using the EnrichmentMap tool for Cytoscape. Nodes represent groups of genes with a common biological meaning described by GO terms. The node size is proportional to the number of genes with a given biological meaning in the transcriptome. The node color reflects the statistical significance level (p-value) for GO term enrichment, and the node outline green color indicates the GO term category ("molecular function"). An edge connects nodes that share a portion of genes. The edge thickness is proportional to the number of shared genes between nodes for the entire transcriptome;

(B) The annotations of various unigenes obtained from Swissprot data using the BlastP and BlastX programs.

B

Gene Id	No of Unigenes	GO Annotations	Annotation Id	Blast Annotation Full Name	$Log_{10}(FC)$
TRINITY_DN14695_c0_g3	4	GO:0044183	RBCX1_ARATH	Chaperonin-like RBCX protein 1, chloroplastic {ECO:0000303|PubMed:21922322}	1,231359793
TRINITY_DN16713_c0_g1	2	GO:0044183, GO:0051787	HSP72_SOLLC<br>HSP7N_ARATH	Heat shock cognate 70 kDa protein 2<br>Heat shock 70 kDa protein 18	-0,68428281

ARATH - *Arabidopsis thaliana*, SOLLC - *Solanum lycopersicum*

MEF-CONTROL, A network binding mainly **BIOTIC FACTORS**, **PHYTOHORMONES**, **REDOX BUFER/ANTIOXIDANTS** and **ION TRANSPORT**

A

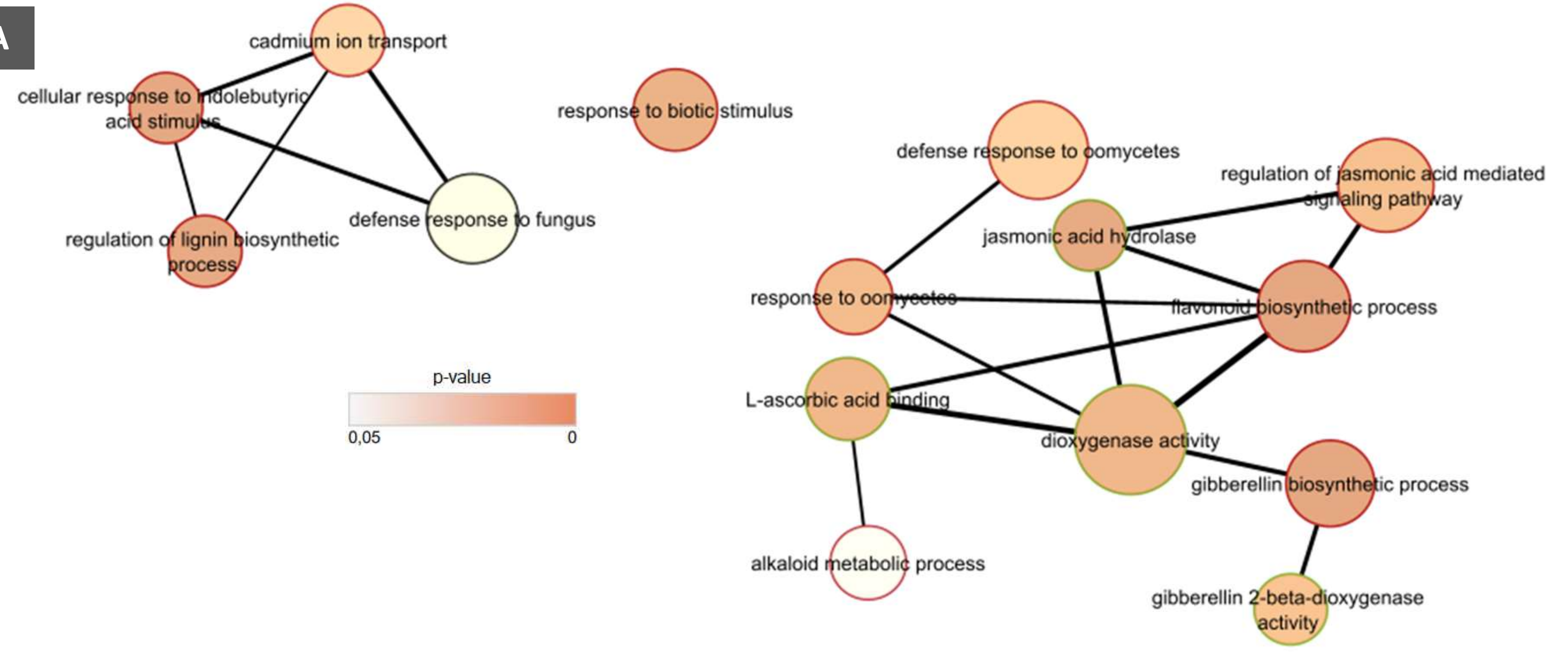

B

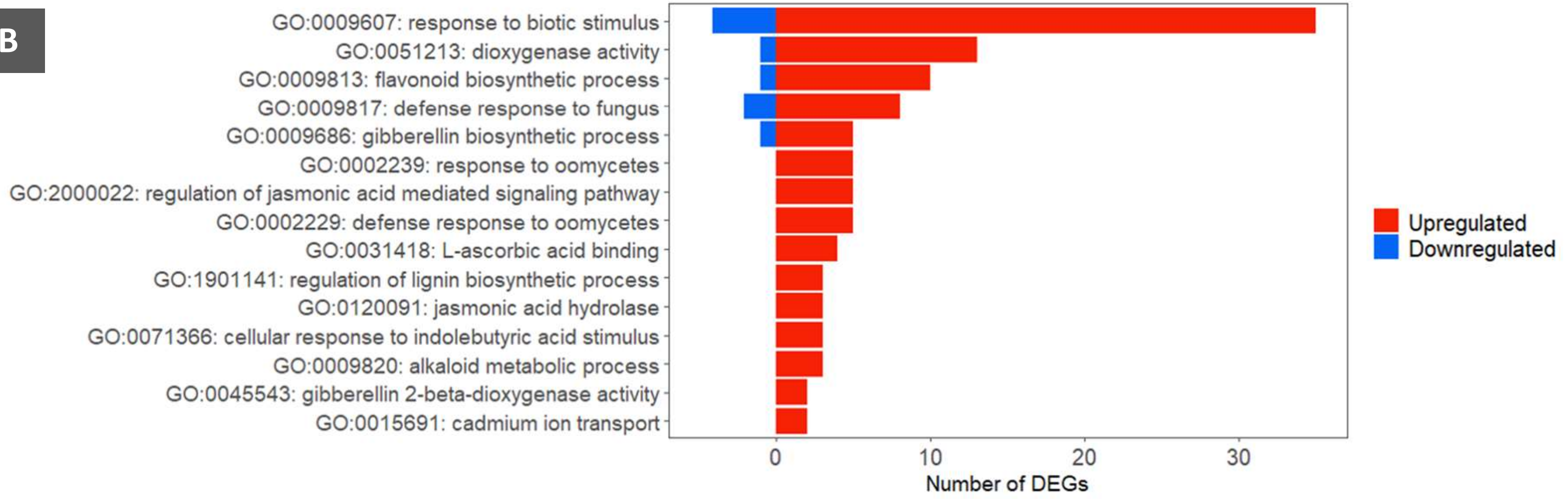

Fig. S7. Changes in the expression profile of genes associated mainly with the biotic factors, phytohormones, redox reactions, and ion transport in MEF-Control comparison.

(A) The gene enrichment map obtained using the EnrichmentMap tool for Cytoscape. Nodes represent groups of genes with a common biological meaning described by GO terms. The node size is proportional to the number of genes with a given biological meaning in the transcriptome. The node color reflects the statistical significance level (p-value) for GO term enrichment, and the node outline color indicates the GO term category for a given node, where green represents “molecular function” terms and red represents “biological process” terms. Edges connect nodes that share a portion of genes. The edge thickness is proportional to the number of shared genes between nodes for the entire transcriptome;

(B) A bar chart showing the number of differentially expressed genes (up- and downregulated) annotated with the analyzed GO terms;

(C) The annotations of various unigenes obtained from Swissprot data using the BlastP and BlastX programs.

MEF-CONTROL, A network binding mainly **BIOTIC FACTORS**, **PHYTOHORMONES**, **REDOX BUFER/ANTIOXIDANTS** and **ION TRANSPORT**

C

Gene Id	No. of Unigenes	GO Annotations	Annotation Id	Blast Annotation Full Name	$Log_{10}(FC)$
TRINITY_DN11826_c3_g3	2	GO:0009607	PTR19_ARATH	Protein NRT1/ PTR FAMILY 4.6	1,306848141
TRINITY_DN3035_c0_g1	1	GO:0009607, GO:0009817	DEF_VIGUN	Defensin-like protein	1,281219463
TRINITY_DN11825_c3_g1	4	GO:0009686	JAMYB_ORYSJ<br>MYB62_ARATH	Transcription factor JAMYB {ECO:0000305}<br>Transcription factor MYB62 {ECO:0000303|PubMed:9839469}	1,245652215
TRINITY_DN11422_c3_g1	7	GO:0009607	B120_ARATH<br>CRK40_ARATH<br>CRK41_ARATH<br>Y1639_ARATH<br>Y4960_ARATH	G-type lectin S-receptor-like serine/threonine-protein kinase B120<br>Cysteine-rich receptor-like protein kinase 40<br>Cysteine-rich receptor-like protein kinase 41<br>G-type lectin S-receptor-like serine/threonine-protein kinase At1g61390<br>Putative receptor-like protein kinase At4g00960	1,203252712
TRINITY_DN17022_c0_g2	5	GO:0009820	DXMT1_COFAR<br>MXMT1_COFAR<br>MXMT2_COFAR<br>XMT1_COFAR<br>XMT1_COFCA	3,7-dimethylxanthine N-methyltransferase<br>Monomethylxanthine methyltransferase 1<br>Monomethylxanthine methyltransferase 2<br>7-methylxanthosine synthase 1<br>7-methylxanthosine synthase 1	1,163336014
TRINITY_DN12966_c4_g5	2	GO:0031418	ACCO_PRUMU	1-aminocyclopropane-1-carboxylate oxidase	1,155471935
TRINITY_DN4012_c0_g1	2	GO:0009607	CE101_ARATH<br>Y1661_ARATH	G-type lectin S-receptor-like serine/threonine-protein kinase CES101<br>Putative G-type lectin S-receptor-like serine/threonine-protein kinase At1g61610	1,104370345
TRINITY_DN14909_c2_g2	3	GO:0009607, GO:0009813	DFRA_ANTMA<br>SNL6_ORYSJ	Dihydroflavonol 4-reductase<br>Cinnamoyl-CoA reductase-like SNL6 {ECO:0000305}	1,089810199
TRINITY_DN16458_c2_g1	1	GO:0009607, GO:0009817	EIX2_SOLLC	Receptor-like protein EIX2 {ECO:0000305}	1,054644953
TRINITY_DN10361_c0_g3	1	GO:0009813	HMOX1_ARATH	Heme oxygenase 1, chloroplastic	1,029512834
TRINITY_DN11825_c3_g4	2	GO:0009686	MYB2_ORYSJ<br>MYB62_ARATH	Transcription factor MYB2 {ECO:0000305}<br>Transcription factor MYB62 {ECO:0000303|PubMed:9839469}	1,026702132
TRINITY_DN10726_c0_g6	2	GO:0009607	PRS2_SOLTU	Pathogenesis-related protein STH-2	0,974398503
TRINITY_DN9608_c0_g1	3	GO:0009607, GO:0009817	DEF07_ARATH<br>DF322_SOLTU	Defensin-like protein 7<br>Defensin-like protein P322	0,962840132
TRINITY_DN9093_c0_g2	3	GO:0009607, GO:2000022	WRK11_ARATH	Probable WRKY transcription factor 11	0,952258656
TRINITY_DN12295_c0_g1	2	GO:0009686, GO:0045543, GO:0051213	G2OX_PHACN	Gibberellin 2-beta-dioxygenase	0,946599091
TRINITY_DN11509_c3_g2	8	GO:0009607	AAP1_ARATH<br>AAP4_ARATH<br>AAP7_ARATH	Amino acid permease 1<br>Amino acid permease 4<br>Probable amino acid permease 7	0,922184406
TRINITY_DN11447_c0_g1	3	GO:0009607	NHL1_ARATH	NDR1/HIN1-like protein 1 {ECO:0000303|Ref.1}	0,908397703
TRINITY_DN18068_c11_g8	1	GO:0009813, GO:0051213, GO:0120091, GO:2000022	DIOX5_ARATH	Probable 2-oxoglutarate-dependent dioxygenase At5g05600 {ECO:0000305}	0,905860858
TRINITY_DN16190_c1_g7	1	GO:1901141	AB29G_ARATH	ABC transporter G family member 29	0,880587706

ARATH - *Arabidopsis thaliana*
VIGUN - *Vigna unguiculata*
ORYSJ - *Oryza sativa*
COFAR - *Coffea arabica*
PRUMU - *Prunus mume*
ANTMA - *Antirrhinum majus*
SOLLC - *Solanum lycopersicum*
SOLTU - *Solanum tuberosum*
PHACN - *Phaseolus coccineus*

MEF-CONTROL, A network binding mainly **BIOTIC FACTORS**, **PHYTOHORMONES**, **REDOX BUFER/ANTIOXIDANTS** and **ION TRANSPORT**

C

Gene Id	No. of Unigenes	GO Annotations	Annotation Id	Blast Annotation Full Name	Log_{10}(FC)
TRINITY_DN15797_c3_g3	3	GO:0051213	SRG1_ARATH	Protein SRG1	0,833529
TRINITY_DN11626_c3_g1	7	GO:0009813, GO:0031418, GO:0051213, GO:0120091, GO:2000022	DIOX5_ARATH<br>DIOX6_ARATH<br>FL3H_ARATH	Probable 2-oxoglutarate-dependent dioxygenase At5g05600 {ECO:0000305}<br>Probable 2-oxoglutarate-dependent dioxygenase At3g111800 {ECO:0000305}<br>Naringenin,2-oxoglutarate 3-dioxygenase	0,822504
TRINITY_DN12295_c1_g2	6	GO:0009686, GO:0045543, GO:0051213	G2OX1_PEA	Gibberellin 2-beta-dioxygenase 1	0,812399
TRINITY_DN12910_c1_g3	16	GO:0002229, GO:0002239, GO:0009607, GO:0009686	CTR1_ARATH<br>EDR1_ARATH<br>SIS8_ARATH	Serine/threonine-protein kinase CTR1 {ECO:0000303|PubMed:8431946}<br>Serine/threonine-protein kinase EDR1<br>Probable serine/threonine-protein kinase SIS8 {ECO:0000305}	0,809017
TRINITY_DN8011_c0_g1	1	GO:0009607	M2K9_ARATH	Mitogen-activated protein kinase kinase 9	0,798203
TRINITY_DN7760_c0_g1	4	GO:0009813	UFOG5_MANES	Anthocyanidin 3-O-glucosyltransferase 5	0,794122
TRINITY_DN11477_c5_g1	4	GO:0002229, GO:0002239, GO:0009607, GO:0009813, GO:0051213	DMR6_ARATH<br>F6H1_ARATH	Protein DOWNY MILDEW RESISTANCE 6 {ECO:0000303|PubMed:15986928}<br>Feruloyl CoA ortho-hydroxylase 1	0,787614
TRINITY_DN12336_c3_g1	4	GO:0009607, GO:0009817	EML3_ARATH<br>SDH7B_ARATH	Protein EMSY-LIKE 3 {ECO:0000303|PubMed:21830950}<br>Succinate dehydrogenase subunit 7B, mitochondrial {ECO:0000305}	0,78747
TRINITY_DN12557_c1_g2	1	GO:0009607	RLK7_ARATH	Receptor-like protein kinase 7 {ECO:0000303|PubMed:20811905}	0,762288
TRINITY_DN14212_c4_g3	7	GO:0051213	JMJ30_ARATH	Lysine-specific demethylase JMJ30 {ECO:0000303|PubMed:18713399}	0,759425
TRINITY_DN13998_c0_g1	31	GO:0009607	AGO10_ARATH<br>AGO1_ARATH<br>PNH1_ORYSJ	Protein argonaute 10<br>Protein argonaute 1<br>Protein argonaute PNH1	0,745957
TRINITY_DN10905_c0_g3	6	GO:0009820, GO:0031418, GO:0051213	ACCH1_ARATH<br>ACCH3_ARATH<br>DV4H_CATRO	1-aminocyclopropane-1-carboxylate oxidase homolog 1<br>1-aminocyclopropane-1-carboxylate oxidase homolog 3<br>Deacetoxyvindoline 4-hydroxylase {ECO:0000303|PubMed:9290645}	0,740305
TRINITY_DN12858_c0_g4	2	GO:0009607, GO:0009817	HEVE_HEVBR	Pro-hevein	0,740094
TRINITY_DN12410_c3_g1	12	GO:0051213	LOX15_SOLTU<br>LOX16_SOLTU	Probable linoleate 9S-lipoxygenase 5<br>Linoleate 9S-lipoxygenase 6	0,733778
TRINITY_DN14194_c2_g2	4	GO:0009607, GO:0015691, GO:0071366	AB35G_ARATH<br>AB36G_ARATH<br>AB42G_ORYSJ	ABC transporter G family member 35<br>ABC transporter G family member 36<br>ABC transporter G family member 42 {ECO:0000303|PubMed:18299247}	0,72991
TRINITY_DN15933_c0_g2	9	GO:0009607	SD11_ARATH<br>Y1141_ARATH<br>Y1150_ARATH<br>Y4729_ARATH	G-type lectin S-receptor-like serine/threonine-protein kinase SD1-1<br>G-type lectin S-receptor-like serine/threonine-protein kinase At1g11410<br>G-type lectin S-receptor-like serine/threonine-protein kinase At1g61500<br>G-type lectin S-receptor-like serine/threonine-protein kinase At4g27290	0,729704
TRINITY_DN13067_c4_g1	3	GO:0009820	MES2_ARATH<br>SABP2_TOBAC	Methylesterase 2<br>Salicylic acid-binding protein 2	0,724933
TRINITY_DN13527_c0_g2	3	GO:0009607	L18B_LUPLU	Protein LlR18B	0,721201

ARATH - *Arabidopsis thaliana*
PEA - *Pisum sativum*
MANES - *Manihot esculenta*
ORYSJ - *Oryza sativa*
CATRO - *Catharanthus roseus*
HEVBR - *Hevea brasiliensis*
SOLTU - *Solanum tuberosum*
TOBAC - *Nicotiana tabacum*
LUPLU - *Lupinus luteus*

MEF-CONTROL, A network binding mainly **BIOTIC FACTORS**, **PHYTOHORMONES**, **REDOX BUFER/ANTIOXIDANTS** and **ION TRANSPORT**

Gene Id	No. of Unigenes	GO Annotations	Annotation Id	Blast Annotation Full Name	$Log_{10}(FC)$
TRINITY_DN12212_c1_g1	9	GO:0009607	MLO12_ARATH ; MLO2_ARATH ; MLO6_ARATH<br>MLOL_LINUS	MLO-like protein 12 ; MLO-like protein 2 ; MLO-like protein 6<br>MLO-like protein	0,716869
TRINITY_DN17761_c0_g1	40	GO:0071366, GO:1901141	AB29G_ARATH; AB33G_ARATH; AB37G_ARATH<br>AB41G_ORYSJ<br>PDR3_TOBAC	ABC transporter G family member 29 ; 33 ; 37<br>ABC transporter G family member 41 {ECO:0000303|PubMed:18299247}<br>Pleiotropic drug resistance protein 3	0,701215
TRINITY_DN12619_c0_g4	5	GO:0002229, GO:0002239, GO:0009607, GO:0009813, GO:0051213	DMR6_ARATH	Protein DOWNY MILDEW RESISTANCE 6 {ECO:0000303|PubMed:15986928}	0,697597
TRINITY_DN11177_c3_g1	3	GO:0009607	Y1743_ARATH	Probable LRR receptor-like serine/threonine-protein kinase At1g74360	0,697047
TRINITY_DN16125_c1_g1	6	GO:0009607	PRPX_HORVU	Pathogen-related protein	0,694831
TRINITY_DN11236_c1_g1	15	GO:0009607	CNG20_ARATH<br>PGLR_VITVI<br>PRF1_SOLLC	Probable cyclic nucleotide-gated ion channel 20, chloroplastic<br>Probable polygalacturonase<br>36.4 kDa proline-rich protein	0,686945
TRINITY_DN15151_c1_g8	1	GO:0051213	LOX15_SOLTU	Probable linoleate 9S-lipoxygenase 5	0,68402
TRINITY_DN10473_c3_g1	4	GO:0009607	PUP10_ARATH ; PUP11_ARATH ; PUP21_ARATH	Probable purine permease 10 ; 11 ; 21	0,68046
TRINITY_DN13054_c1_g1	2	GO:0009607, GO:0009817	EIX2_SOLLC	Receptor-like protein EIX2 {ECO:0000305}	0,675497
TRINITY_DN13600_c0_g1	19	GO:0009813	CHS1_CICAR<br>CHS1_SOYBN ; CHS3_SOYBN<br>CHS1_TRISU ; CHS2_TRISU ; CHS4_TRISU<br>CHS4_MEDSA ; CHS9_MEDSA<br>CHS4_PEA ; CHSB_PEA<br>CHSY_PHAVU<br>CHSY_PUEML	Chalcone synthase 1<br>Chalcone synthase 1 ; Chalcone synthase 3<br>Chalcone synthase 1 ; Chalcone synthase 2 ; Chalcone synthase 4<br>Chalcone synthase 4 ; Chalcone synthase 9<br>Chalcone synthase 4 ; Chalcone synthase 1B<br>Chalcone synthase 17<br>Chalcone synthase	0,673534
TRINITY_DN17733_c1_g2	6	GO:0009607	AMT23_ORYSJ<br>AMT2_ARATH<br>AMT31_ORYSJ	Ammonium transporter 2 member 3<br>Ammonium transporter 2<br>Ammonium transporter 3 member 1	0,673116
TRINITY_DN2291_c0_g1	1	GO:0002229, GO:0002239, GO:0009607	LSU2_ARATH	Protein RESPONSE TO LOW SULFUR 2 {ECO:0000303|PubMed:25628631}	0,64529
TRINITY_DN13561_c3_g1	12	GO:0009607, GO:0009817, GO:0051213	DOX1_ARATH	Alpha-dioxygenase 1	0,639462
TRINITY_DN8465_c0_g2	19	GO:0009607	DSC2_ARATH<br>TAO1_ARATH<br>TMVRN_NICGU	Disease resistance-like protein DSC2 {ECO:0000305}<br>Disease resistance protein TAO1 {ECO:0000305}<br>TMV resistance protein N	0,629417
TRINITY_DN13476_c1_g1	5	GO:0009813	RNLE_SOLLC<br>RNS1_ARATH	Extracellular ribonuclease LE<br>Ribonuclease 1	0,625693
TRINITY_DN11626_c3_g6	2	GO:0002229, GO:0002239, GO:0009607, GO:0009813, GO:0031418, GO:0051213, GO:0120091, GO:2000022	DIOX5_ARATH<br>DLO1_ARATH<br>FL3H_PETCR	Probable 2-oxoglutarate-dependent dioxygenase At5g05600 {ECO:0000305}<br>Protein DMR6-LIKE OXYGENASE 1 {ECO:0000303|PubMed:25376907}<br>Flavanone 3-dioxygenase	0,620701
TRINITY_DN13238_c0_g1	3	GO:0009607, GO:0015691, GO:0071366, GO:1901141	AB29G_ARATH<br>AB36G_ARATH	ABC transporter G family member 29<br>ABC transporter G family member 36	0,6207
TRINITY_DN445_c0_g1	2	GO:0009607, GO:0009817	PER21_ARATH	Peroxidase 21	0,62025
TRINITY_DN8148_c0_g1	3	GO:2000022	MTP10_ARATH	Metal tolerance protein 10	0,605338

C

ARATH - *Arabidopsis thaliana*
LINUS - *Linum usitatissimum*
ORYSJ - *Oryza sativa*
TOBAC - *Nicotiana tabacum*
HORVU - *Hordeum vulgare*
VITVI - *Vitis vinifera*
SOLLC - *Solanum lycopersicum*
SOLTU - *Solanum tuberosum*
CICAR - Cicer *arietinum*
SOYBN - *Glycine max*
TRISU - *Trifolium subterraneum*
MEDSA - *Medicago sativa*
PEA - *Pisum sativum*
PHAVU - *Phaseolus vulgaris*
PUEML - *Pueraria montana* var. lobata
NICGU - *Nicotiana glutinosa*
PETCR - *Petroselinum crispum*

MEF-CONTROL, A network binding mainly **BIOTIC FACTORS**, **PHYTOHORMONES**, **REDOX BUFER/ANTIOXIDANTS** and **ION TRANSPORT**

C

Gene Id	No of Unigenes	GO Annotations	Annotation Id	Blast Annotation Full Name	$Log_{10}(FC)$
TRINITY_DN5885_c0_g1	3	GO:0009686	KSA_PEA	*Ent*-copalyl diphosphate synthase, chloroplastic	-0,67023895
TRINITY_DN11438_c2_g4	7	GO:0009607	AAP1_ARATH<br>AAP6_ARATH<br>AAP8_ARATH	Amino acid permease 1<br>Amino acid permease 6<br>Amino acid permease 8	-0,71275331
TRINITY_DN13561_c3_g2	9	GO:0009607, GO:0009817, GO:0051213	DOX1_ARATH	Alpha-dioxygenase 1	-0,76090486
TRINITY_DN15575_c6_g2	7	GO:0009607, GO:0009817	CER1_ARATH<br>CERL1_ARATH<br>GLO15_ORYSI<br>GLO17_ORYSJ	Very-long-chain aldehyde decarbonylase CER1 {ECO:0000305}<br>Protein CER1-like 1<br>Very-long-chain aldehyde decarbonylase GL1-5 {ECO:0000305}<br>Very-long-chain aldehyde decarbonylase GL1-7 {ECO:0000305}	-0,8022464
TRINITY_DN15999_c1_g2	5	GO:0009813	AA5GT_DIACA<br>BGL11_ARATH	Cyanidin 3-O-glucoside 5-O-glucosyltransferase (acyl-glucose)<br>Beta-glucosidase 11	-1,11518497
TRINITY_DN10461_c0_g1	4	GO:0009607	AGAL_COFAR<br>AGAL_ORYSJ<br>MEL_ZYGMR	Alpha-galactosidase<br>Alpha-galactosidase<br>Alpha-galactosidase	-1,63492909

PEA - *Pisum sativum*
ARATH - *Arabidopsis thaliana*
ORYSJ - *Oryza sativa*
DIACA - *Dianthus caryophyllus*
COFAR - *Coffea arabica*
ZYGMR - *Zygotorulaspora mrakii*

MEF-CONTROL, A network binding mainly **RHYTHMIC PROCESSES**, **PHYTOHORMONES**, **POLYSACCHARIDES**, and **WATER DEPRIVATION**

A

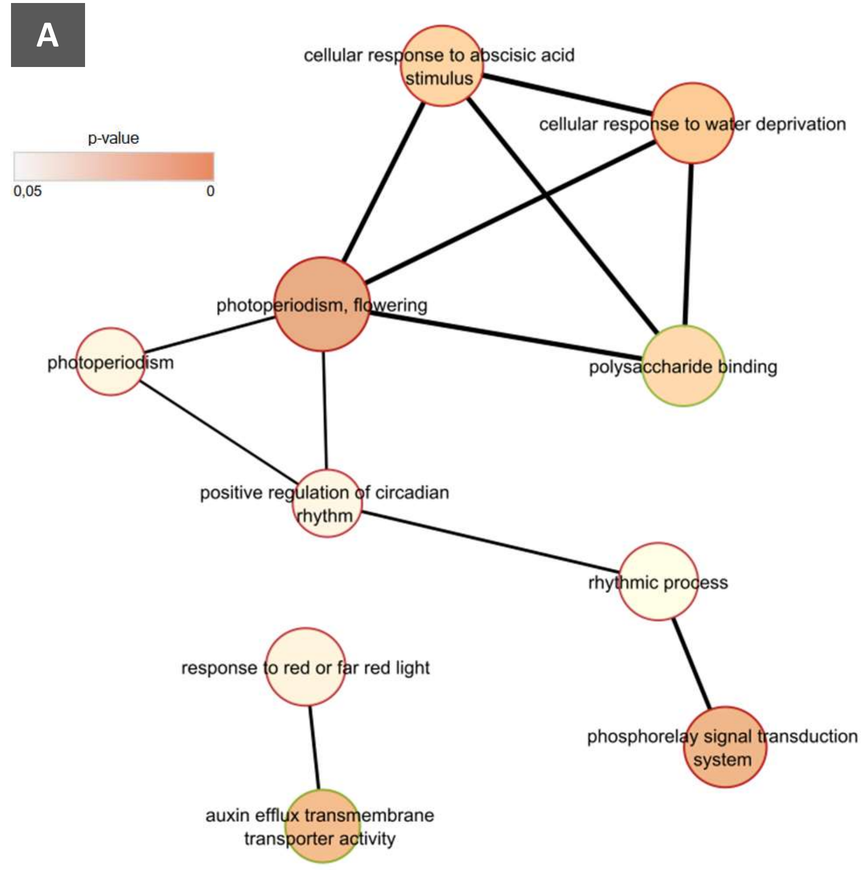

Fig. S8. Changes in the expression profile of genes associated mainly with the rhythmic processes, phytohormones, polysaccharides, and water deprivation in MEF-Control comparison.

(A) The gene enrichment map obtained using the EnrichmentMap tool for Cytoscape. Nodes represent groups of genes with a common biological meaning described by GO terms. The node size is proportional to the number of genes with a given biological meaning in the transcriptome. The node color reflects the statistical significance level (p-value) for GO term enrichment, and the node outline color indicates the GO term category for a given node, where green represents “molecular function” terms and red represents “biological process” terms. Edges connect nodes that share a portion of genes. The edge thickness is proportional to the number of shared genes between nodes for the entire transcriptome;

(B) A bar chart showing the number of differentially expressed genes (up- and downregulated) annotated with the analyzed GO terms;

(C) The annotations of various unigenes obtained from Swissprot data using the BlastP and BlastX programs.

B

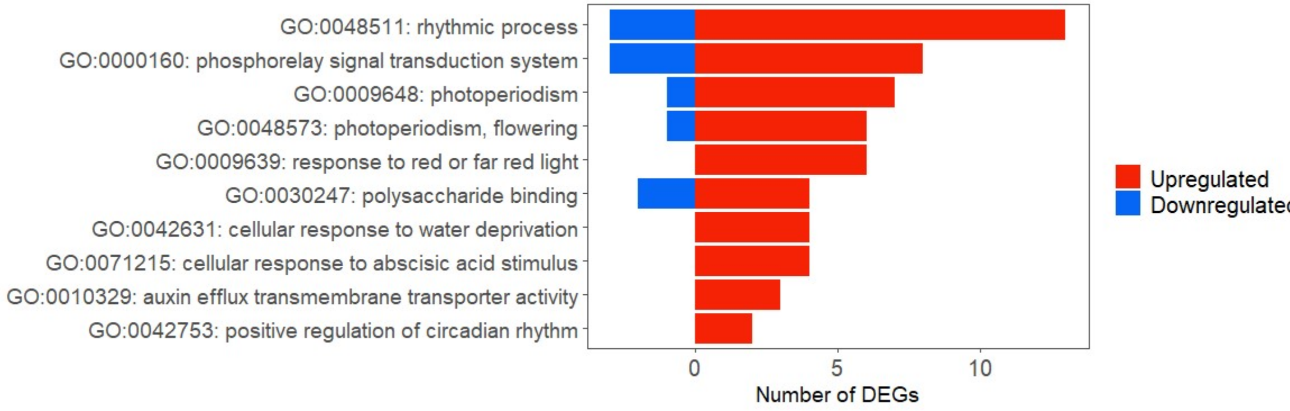

MEF-CONTROL, A network binding mainly **RHYTMIC PROCESSES**, **PHYTOHORMONES**, **POLYSACCHARIDES**, and **WATER DEPRIVATION**

C

Gene Id	No. of Unigenes	GO Annotations	Annotation Id	Blast Annotation Full Name	Log_{10}(FC)
TRINITY_DN13747_c2_g6	2	GO:0010329	AB11B_ARATH<br>AB4B_ARATH	ABC transporter B family member 11<br>ABC transporter B family member 4	1,460926673
TRINITY_DN12648_c2_g3	52	GO:0009648, GO:0048573	BFT_ARATH<br>FT_ARATH<br>HD3A_ORYSJ	Protein BROTHER of FT and TFL 1<br>Protein FLOWERING LOCUS T {ECO:0000303|PubMed:10583960}<br>Protein HEADING DATE 3A	1,404018364
TRINITY_DN13747_c2_g3	1	GO:0010329	AB11B_ARATH	ABC transporter B family member 11	1,207452029
TRINITY_DN15526_c3_g2	4	GO:0009648, GO:0030247, GO:0042631, GO:0048573, GO:0071215	LRL11_ARATH<br>LRL12_ARATH	LEAF RUST 10 DISEASE-RESISTANCE LOCUS RECEPTOR-LIKE PROTEIN KINASE-like 1.1 {ECO:0000303|PubMed:12805585}<br>LEAF RUST 10 DISEASE-RESISTANCE LOCUS RECEPTOR-LIKE PROTEIN KINASE-like 1.2 {ECO:0000303|PubMed:12805585}	1,162238627
TRINITY_DN6849_c0_g1	1	GO:0009639	GIL1_ARATH	Protein GRAVITROPIC IN THE LIGHT 1 {ECO:0000303|PubMed:16640600}	1,15640116
TRINITY_DN14278_c3_g2	18	GO:0048511	COL10_ARATH<br>COL9_ARATH	Zinc finger protein CONSTANS-LIKE 10<br>Zinc finger protein CONSTANS-LIKE 9	1,070488389
TRINITY_DN13268_c1_g8	1	GO:0000160, GO:0048511	APRR5_ARATH	Two-component response regulator-like APRR5	1,066685863
TRINITY_DN13268_c2_g1	1	GO:0000160, GO:0048511	APRR5_ARATH	Two-component response regulator-like APRR5	1,016567706
TRINITY_DN11267_c6_g5	1	GO:0048511	ADO3_ARATH	Adagio protein 3	0,998629016
TRINITY_DN11988_c1_g2	1	GO:0000160	EF112_ARATH	Ethylene-responsive transcription factor ERF112	0,966640475
TRINITY_DN10909_c0_g9	2	GO:0009648, GO:0042753, GO:0048511	EF4L4_ARATH	Protein ELF4-LIKE 4	0,92127674
TRINITY_DN18030_c5_g1	2	GO:0030247	LRL11_ARATH	LEAF RUST 10 DISEASE-RESISTANCE LOCUS RECEPTOR-LIKE PROTEIN KINASE-like 1.1 {ECO:0000303|PubMed:12805585}	0,912486744
TRINITY_DN16750_c1_g2	4	GO:0000160	EF118_ARATH	Ethylene-responsive transcription factor ERF118	0,889138746
TRINITY_DN11899_c1_g1	8	GO:0009648, GO:0030247, GO:0042631, GO:0048573, GO:0071215	LRL12_ARATH<br>LRL14_ARATH<br>LRL21_ARATH	LEAF RUST 10 DISEASE-RESISTANCE LOCUS RECEPTOR-LIKE PROTEIN KINASE-like 1.2 {ECO:0000303|PubMed:12805585}<br>LEAF RUST 10 DISEASE-RESISTANCE LOCUS RECEPTOR-LIKE PROTEIN KINASE-like 1.4 {ECO:0000303|PubMed:12805585}<br>LEAF RUST 10 DISEASE-RESISTANCE LOCUS RECEPTOR-LIKE PROTEIN KINASE-like 2.1 {ECO:0000303|PubMed:12805585}	0,871479302
TRINITY_DN17516_c6_g1	5	GO:0000160, GO:0048511	APRR5_ARATH<br>APRR9_ARATH	Two-component response regulator-like APRR5<br>Two-component response regulator-like APRR9	0,859921231
TRINITY_DN11267_c6_g6	1	GO:0048511	ADO3_ARATH	Adagio protein 3	0,855145745
TRINITY_DN15943_c4_g1	3	GO:0009639, GO:0009648, GO:0042753, GO:0048511, GO:0048573	ELF4_ARATH	Protein EARLY FLOWERING 4	0,820340202
TRINITY_DN12259_c1_g4	7	GO:0009639	PIF3_ARATH<br>PIL15_ORYSJ	Transcription factor PIF3<br>Transcription factor PHYTOCHROME INTERACTING FACTOR-LIKE 15 {ECO:0000303|PubMed:17485859}	0,814491582
TRINITY_DN12910_c1_g3	16	GO:0000160	CTR1_ARATH<br>EDR1_ARATH<br>SIS8_ARATH	Serine/threonine-protein kinase CTR1 {ECO:0000303|PubMed:8431946}<br>Serine/threonine-protein kinase EDR1<br>Probable serine/threonine-protein kinase SIS8 {ECO:0000305}	0,809016813
TRINITY_DN8011_c0_g1	1	GO:0000160	M2K9_ARATH	Mitogen-activated protein kinase kinase 9	0,798203063

MEF-CONTROL, A network binding mainly **RHYTMIC PROCESSES**, **PHYTOHORMONES**, **POLYSACCHARIDES**, and **WATER DEPRIVATION**

C

Gene Id	No. of Unigenes	GO Annotations	Annotation Id	Blast Annotation Full Name	Log_{10}(FC)
TRINITY_DN10546_c2_g2	4	GO:0009639	HLS1L_ARATH<br>HLS1_ARATH	Probable N-acetyltransferase HLS1-like<br>Probable N-acetyltransferase HLS1	0,796912053
TRINITY_DN14212_c4_g3	7	GO:0048511	JMJ30_ARATH	Lysine-specific demethylase JMJ30 {ECO:0000303|PubMed:18713399}	0,759424663
TRINITY_DN13998_c0_g1	31	GO:0009639	AGO10_ARATH<br>AGO1_ARATH<br>PNH1_ORYSJ	Protein argonaute 10<br>Protein argonaute 1<br>Protein argonaute PNH1	0,7459569
TRINITY_DN12759_c1_g4	2	GO:0009639	GIL1_ARATH	Protein GRAVITROPIC IN THE LIGHT 1 {ECO:0000303|PubMed:16640600}	0,745453144
TRINITY_DN11934_c1_g1	10	GO:0048511	KAT1_ARATH<br>KAT2_ARATH	Potassium channel KAT1<br>Potassium channel KAT2	0,737637812
TRINITY_DN12449_c1_g2	8	GO:0010329	PILS5_ARATH<br>PILS7_ARATH	Protein PIN-LIKES 5 {ECO:0000303|PubMed:22504182}<br>Protein PIN-LIKES 7 {ECO:0000303|PubMed:22504182}	0,709675843
TRINITY_DN14901_c0_g1	1	GO:0009648, GO:0030247, GO:0042631, GO:0048573, GO:0071215	LRL12_ARATH	LEAF RUST 10 DISEASE-RESISTANCE LOCUS RECEPTOR-LIKE PROTEIN KINASE-like 1.2 {ECO:0000303|PubMed:12805585}	0,699881408
TRINITY_DN13268_c1_g1	6	GO:0000160, GO:0048511	APRR5_ARATH<br>PRR95_ORYSJ	Two-component response regulator-like APRR5<br>Two-component response regulator-like PRR95	0,690613011
TRINITY_DN11087_c8_g2	9	GO:0048511	FB348_ARATH	F-box protein At2g16365	0,68409727
TRINITY_DN14775_c2_g1	2	GO:0042631, GO:0071215	U74D1_ARATH<br>U74E2_ARATH	UDP-glycosyltransferase 74D1<br>UDP-glycosyltransferase 74E2	0,66860636
TRINITY_DN445_c0_g1	2	GO:0048511	PER21_ARATH	Peroxidase 21	0,620250034
TRINITY_DN13612_c1_g4	11	GO:0009648, GO:0048573	BH130_ARATH	Transcription factor bHLH130	0,603729255

Gene Id	No. of Unigenes	GO Annotations	Annotation Id	Blast Annotation Full Name	Log_{10}(FC)
TRINITY_DN14251_c0_g1	1	GO:0000160	EF112_ARATH<br>ERF71_ARATH	Ethylene-responsive transcription factor ERF112<br>Ethylene-responsive transcription factor ERF071	-0,60122341
TRINITY_DN8562_c0_g1	3	GO:0009648, GO:0048511, GO:0048573	ELF3_ARATH<br>HD3B_ORYSJ	Protein EARLY FLOWERING 3<br>Protein HEADING DATE 3B	-0,64442339
TRINITY_DN13970_c2_g1	19	GO:0048511	LNK3_ARATH<br>LNK4_ARATH	Protein LNK3 {ECO:0000303|PubMed:23818596}<br>Protein LNK4 {ECO:0000303|PubMed:23818596}	-0,75661843
TRINITY_DN12058_c2_g9	3	GO:0030247	AMYG_ASPOR	Glucoamylase	-0,79515153
TRINITY_DN8063_c0_g1	2	GO:0048511	GAT12_ARATH	GATA transcription factor 12 {ECO:0000303|PubMed:15084732}	-0,842145
TRINITY_DN8273_c0_g1	5	GO:0000160	ARR24_ARATH	Two-component response regulator 24 {ECO:0000312|EMBL:AED93557.1}	-0,87691004
TRINITY_DN7281_c0_g1	1	GO:0000160	CRF4_ARATH	Ethylene-responsive transcription factor CRF4	-0,90659593
TRINITY_DN11545_c2_g1	1	GO:0030247	SSY3_SOLTU	Soluble starch synthase 3, chloroplastic/amyloplastic	-0,98158441

ARATH - *Arabidopsis thaliana*
ORYSJ - *Oryza sativa*
ASPOR - *Aspergillus oryzae*
SOLTU - *Solanum tuberosum*

MEF-CONTROL: SUMMARY AND CONCLUSIONS

Limitation of JA biosynthesis after the opening of pollen chambers affects the metabolism, transport, and homeostasis of numerous organic and inorganic substances, as well as the organization of various structures in *L. luteus* grown under well-watered conditions

The overrepresented and closely associated GO terms related to **carbohydrate metabolism** were: UDP-glycosyltransferase activity, quercetin 3-O-glucosyltransferase activity, and quercetin 7-O-glucosyltransferase activity (Fig. S1A), with a predominance of upregulated DEGs (Fig. S1B, C). UDP-glucosyltransferases participate, among others functions, in the biosynthesis of phytohormone conjugates, which in most cases are biologically inactive, but serve other roles such as transport or storage forms (Ostrowski and Jakubowska 2014).
The remaining GO terms associated with carbohydrate metabolism included gluconeogenesis, sucrose synthase activity, starch binding, β-galactosidase activity, and hexosyltransferase activity (Fig. S1A). All DEGs in the first three GO terms were downregulated (Fig. S1B, C). Considering the functions of the proteins encoded by these genes in other plant species, it can be concluded that the limitation of JA biosynthesis after *L. luteus* anther dehiscence may be associated with the suspension of sugar synthesis. This is also likely related to enhanced sugar degradation, as there was a significant overrepresentation of GO terms (chitinase activity, chitin binding, chitin catabolic process, polysaccharide catabolic process, Fig. S2A) associated with chitinases and pectinesterases (Fig. S2C). In different species, these enzymes are responsible for the breakdown of polysaccharides, and in *L. luteus* - with the exception of one DEG - all corresponding genes were upregulated (Fig. S2B). Plant chitinases were discovered as fungicidal agents; therefore, their action was long considered exclusively in this context. However, apart from chitin, the substrate for chitinases are also ARABINOGALACTAN PROTEINS (AGPs), the hydrolysis of which leads to loosening of the cell wall structure; moreover the chitooligosaccharides formed as a result of this reaction act as signaling molecules that regulate numerous processes (Kisiel et al. 2016).

Further transcriptomic analyses confirmed changes in the expression of genes related to **cell wall modification**. Among the DEGs annotated with the term plant-type cell wall organization (Fig. S3A), there were 5 genes with increased expression encoding: MYB87, associated with cell wall organization and remodelling (Fujiwara et al. 2014); two EXPANSINS-A4 (EXPA4), responsible for loosening and extension of cell walls; and two β-GALACTOSIDASES (BGALs), which hydrolyse terminal β-D-galactose residues in β-D-galactosides (Fig. S3B, C). Among the downregulated DEGs, there were: COBRA-LIKE protein 3/6 (COBL3/6) playing an important role in cellulose deposition, and EXP14/16 (Fig. S3B, C). Three DEGs annotated as EXPs and assigned to the term plant-type cell wall organization were also assigned to the term syncytium formation (Fig. S3A-C). In turn, the term cell wall biogenesis (Fig. S3A) included two upregulated genes encoding XYLOGLUCAN ENDOTRANSGLUCOSYLASE/HYDROLASE PROTEINS (XTHs), which catalyse xyloglucan endohydrolysis, and five downregulated genes encoding: COBL3/6, two catalytic subunits of CELLULOSE SYNTHASE A (CESA1) involved in the primary cell wall formation (Desprez et al. 2007), and two GALACTOSIDE 2-α-L-FUCOSYLTRANSFERASES (FUTs) involved in cell wall biosynthesis (Sarria et al. 2001) (Fig. S3B, C).

The GO terms related to **metabolism and transport of lipids** were: phospholipase C activity, lipase activity and phospholipid transporter activity (Fig. S4A). Among all DEGs, one was downregulated (PHOSPHOLIPID-TRANSPORTING ATPase 3, ALA3, involved in lipid translocation during vegetative growth and reproductive success) (McDowell et al. 2013); the rest were upregulated, including two NON-SPECIFIC PHOSPHOLIPASE C3/4 (NPC3/4), which may play a role in signal transduction and the synthesis of storage lipids, three GDSL ESTERASE/LIPASE (EXL2/3;GDL87/90) connected with lipid degradation, and METHYLESTERASE 2 (MES2) which may be related to the formation of methyl derivatives of phytohormones (Fig. S4B, C).

MEF-CONTROL: SUMMARY AND CONCLUSIONS

In the MEF-Control comparison, there were also many enriched GO terms associated with **various types of transport** (Fig. S5).

One of the most important was **amino acid transport**, and DEGs - annotated mainly as AMINO ACID PERMEASES (AAPs) and PROLINE/LYSINE HISTIDINE TRANSPORTERs (PROTs/LHTs) (Fig. S5C1) - were assigned to the GOs: L-proline transmembrane transporter activity, amino acid transmembrane transport, L-glutamate transmembrane transporter activity, and amino acid transmembrane transporter activity (Fig. S5A, B).

Overrepresented GO term - **symporter activity** (Fig. S5A), containing 14 up- and down-regulated DEGs (Fig. S5B, C2), enabled active transport of a solute across a membrane by a mechanism in which two or more species were transported together in the same direction. The substances transported via symport route were sulfates, polyols, amino acids, and phosphates; whereas sulfates and boron were **transported via antiport** (Fig. S5C3; GO: anion:anion antiporter activity, Fig. S5A, B), which allowed the solutes to be transferred from one side of a membrane to the other.

In the case of **water transport** (GO: water channel activity, Fig. S5A), the expression of two genes annotated as AQUAPORIN TIP1-3 (TIP13) was negatively regulated, while gene encoding AQUAPORIN NIP5-1 (NIP51) was positively regulated (Fig. S5B, C4).

Other DEGs belonging to the GO term: **channel activity** and **borate transport** (Fig. S5A) were related to the transport of malates, potassium, ammonium, boron, and calcium (Fig. S5B, C4, C5).

Significant overrepresentation of two **protein folding** terms: protein folding chaperone and misfolded protein binding (Fig. S6A) was found. There was a 17-fold increase in expression relative to the control for the gene encoding CHAPERONIN-LIKE RBCX PROTEIN1 (RBCX1), and a 5-fold decrease in the expression of the gene annotated as HEAT SHOCK PROTEIN (HSP7N) (Fig. S6B).

We identified also a large **network** of interconnections between overrepresented GO terms related to: biotic factors (fungus, oomycetes), phytohormones (JAs, GAs, auxins), redox reactions/antioxidants (L-ascorbic acid), and ion transport (cadmium) (Fig. S7A-C).

The **second similar network** included GO terms related to: rhythmic processes, photoperiodism, phytohormones (auxins), polysaccharides, and water deprivation (Fig. S8A-C). The vast majority of DEGs assigned to important GO terms were upregulated in both networks.

The function of the predicted proteins/enzymes/transcription factors to which the individual DEGs identified in *L. luteus* were annotated was determined primarily based on the functions of these proteins found in *A. thaliana* but also in other plant species.

MEF-CONTROL: REFERENCES

Ostrowski M and Jakubowska A (2014) UDP-glycosyltransferases of plant hormones. Adv Cell Biol 41:271–288. https://doi.org/10.2478/acb-2014-0003

Kisiel A, Jęckowska K, Kępczyńska E (2016) The role of chitinases in plant development. Adv Cell Biol 43:273–287

Fujiwara S, Mitsuda N, Nakai Y, Kigoshi K, Suzuki K, Ohme-Takagi M (2014) Chimeric repressor analysis identifies MYB87 as a possible regulator of morphogenesis via cell wall organization and remodeling in *Arabidopsis*. Biotechnol Lett 36:1049–1057. https://doi.org/10.1007/s10529-013-1451-8

Desprez T, Juraniec M, Crowell EF, Jouy H, Pochylova Z, Parcy F, Höfte H, Gonneau M, Vernhettes S (2007) Organization of cellulose synthase complexes involved in primary cell wall synthesis in *Arabidopsis thaliana*. Proc Natl Acad Sci USA 104:15572–15577. https://doi.org/10.1073/pnas.0706569104

Sarria R, Wagner TA, O'Neill MA, Faik A, Wilkerson CG, Keegstra K, Raikhel NV (2001) Characterization of a family of *Arabidopsis* genes related to xyloglucan fucosyltransferase 1. Plant Physiol 127:1595–1606. https://doi.org/10.1104/pp.010410

McDowell SC, López-Marqués RL, Poulsen LR, Jorgensen M, Vestergaard AL, Maeda Y, Kato N, Palmgren MG (2013) Loss of the *Arabidopsis thaliana* P_4-ATPase ALA3 reduces adaptability to temperature stresses and impairs vegetative, pollen, and ovule development. PLoS One 8:e62577. https://doi.org/10.1371/journal.pone.0062577

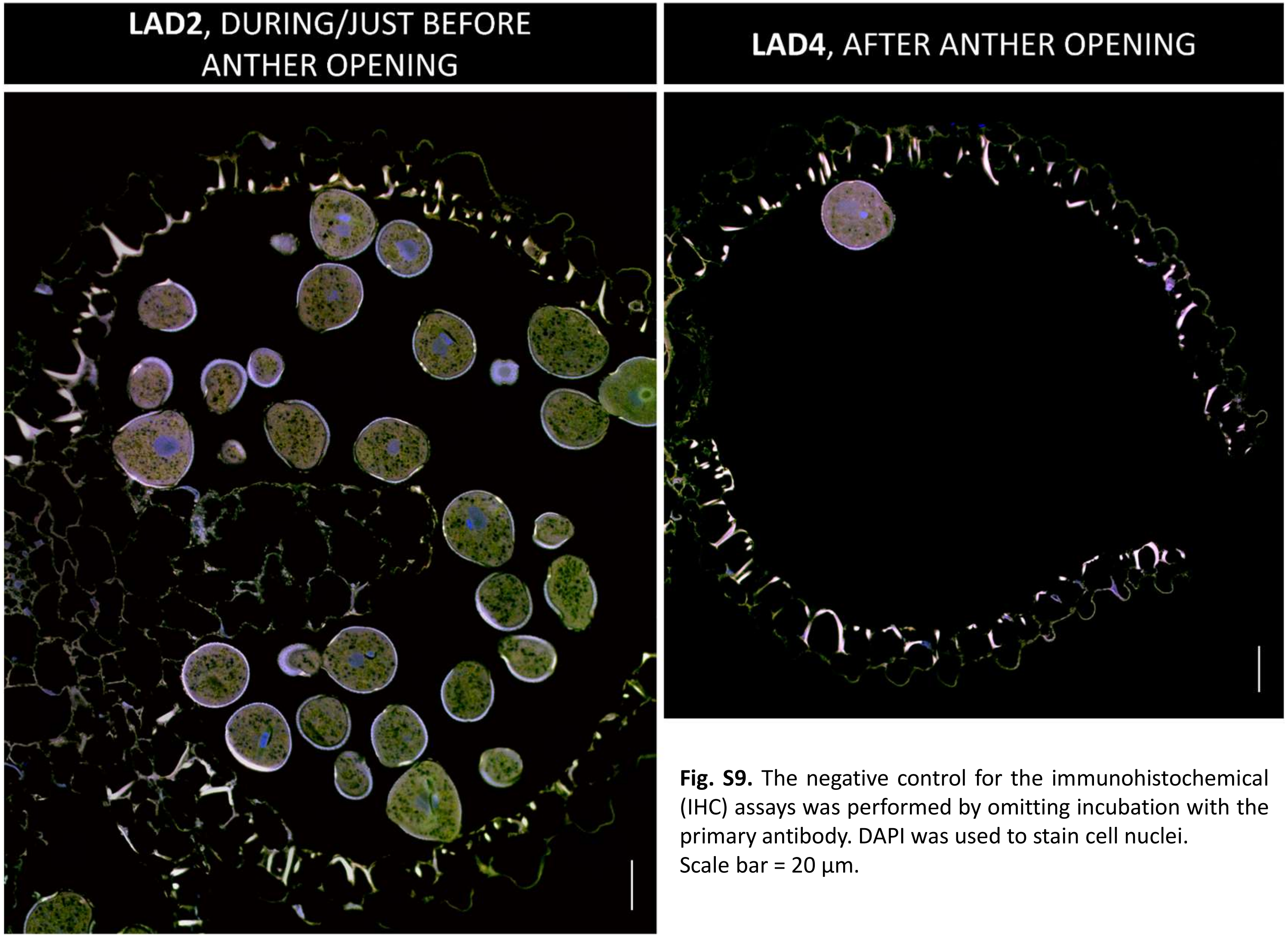

Fig. S9. The negative control for the immunohistochemical (IHC) assays was performed by omitting incubation with the primary antibody. DAPI was used to stain cell nuclei.
Scale bar = 20 µm.

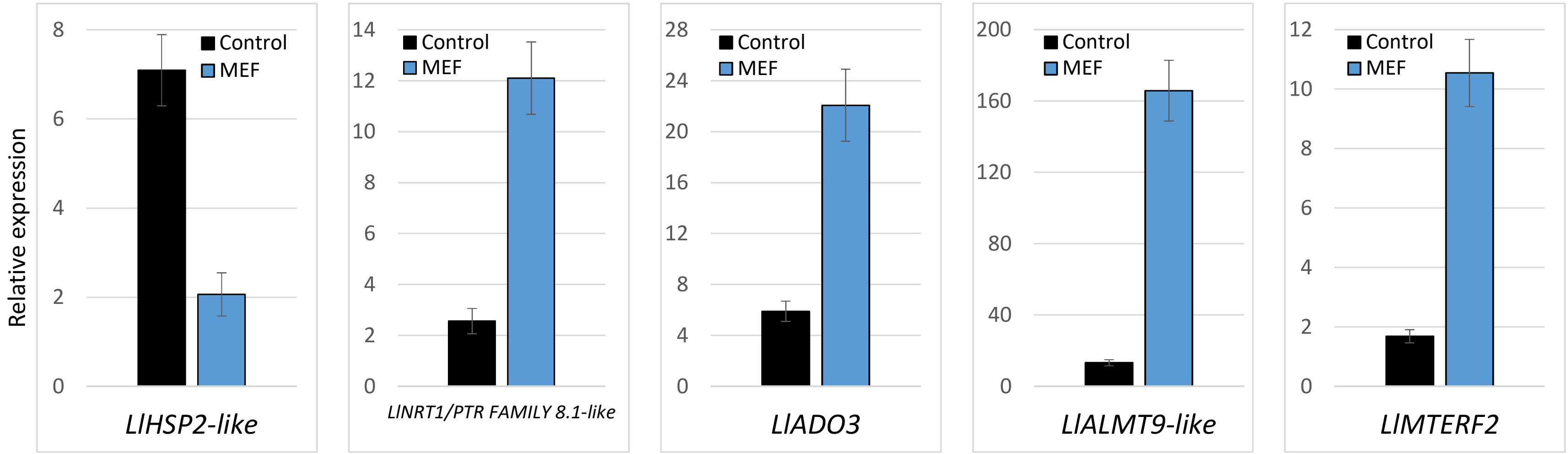

Fig. S10. qPCR validation of differential expression patterns of selected genes. The relative quantification of the gene expression level was determined using *LlACT* (*ACTIN)* as a reference gene.

LlHSP2-like – Heat shock cognate 70 kDa protein 2, mRNA. *Lupinus luteus* predicted protein sequence was annotated as HSP72_SOLLC (*Solanum lycopersicum*) in BlastX and HSP7N_ARATH (*Arabidopsis thaliana*) in BlastP; the gene name is *HSC-2*;

LlNRT1/PTR FAMILY 8.1-like – Nitrate transporter 1/Peptide transporter family 8.1-like, mRNA. *Lupinus luteus* predicted protein sequence was annotated as PTR5_ARATH or PTR1_ARATH in BlastX and BlastP; the gene name is *AtNPF8.2*;

LlADO3 – Adagio protein 3, mRNA. *Lupinus luteus* predicted protein sequence was annotated as ADO3_ARATH in BlastX and BlastP;

LlALMT9-like – Aluminum-activated malate transporter 9-like, mRNA. *Lupinus luteus* predicted protein sequence was annotated as ALMT9_ARATH in BlastX and ALMT9_ARATH/ ALMT3_ARATH in BlastP;

LlMTERF2 – Transcription termination factor MTERF2, mRNA. *Lupinus luteus* predicted protein sequence was annotated as MTER2_ARATH in BlastX and BlastP.

Table S1. Sequences of gene specific primers and numbers of UNIVERSAL PROBE LIBRARY (UPL) probes used in qPCRs for genes connected with biosynthesis and perception of jasmonates (JAs). The qPCR programs were also presented, when UPL probe or SYBR GREEN dye were used as the fluorescence detection system.

LlAOS *- ALLENE OXIDE SYNTHASE*; ***LlAOC*** *- ALLENE OXIDE CYCLASE*; ***LlOPR3*** *- OPDA REDUCTASE 3*; ***LlCOI1*** *- CORONATINE INSENSITIVE 1.*

		LlAOS		***LlAOC***		***LlOPR3***		***LlCOI1***	
**GENE SPECIFIC PRIMERS**		FP: GAGGTATTTGTGGCTCGTAGGT		FP: TTCACGATTTGCCAACTGAG		FP: CTACCTGACACGGGCTTTCC		FP: GAACCCTCCCCAATTCTCCG	
		RP: CCATTACTCCAAACCACATGC		RP: CAGCCAGAGATGGTTCAACA		RP: AACTCTGGCATGTTGGTCGT		RP: ACCTGGAGTCGTTGAAGCTG	
**FLUORESCENCE DETECTION SYSTEM**		Hydrolysis probe		Hydrolysis probe		SYBR Green dye		SYBR Green dye	
**UPL PROBE NO**		9		139		-		-	
**qPCR PROGRAM:**									
**PRE-INCUBATION** x 1 cycle	Ramp rate 20 °C/s	95 °C	600 s	95 °C	600 s	95 °C	600 s	95 °C	600 s
**AMPLIFICATION** x 45 cycles	DENATURATION	95 °C	10 s	95 °C	10 s	95 °C	10 s	95 °C	10 s
	ANNEALING OF PRIMERS	52 °C	15 s	49 °C	15 s	58 °C	10 s	58 °C	10 s
	EXTENSION	72 °C	1 s	72 °C	1 s	72 °C	5 s	72 °C	20 s
**MELTING CURVE** x 1 cycle	20 °C/s					95 °C	0 s	95 °C	0 s
	20 °C/s					65 °C	15 s	65 °C	15 s
	0.1 °C/s continuous					95 °C	0 s	95 °C	0 s
**COOLING** x 1 cycle	20 °C/s	40 °C	30 s	40 °C	30 s	40 °C	30 s	40°C	30 s

Table S2. Sequences of gene specific primers and UNIVERSAL PROBE LIBRARY (UPL) probes numbers used in qPCRs made for validation of differential expression of selected genes (RNA-Seq, MEF-Control Comparison). The qPCR programs were also presented, when UPL probe or SYBR GREEN dye were used as the fluorescence detection system.

LlHSP2-like *– HEAT SHOCK COGNATE 70 kDa PROTEIN 2/HEAT SHOCK PROTEIN 2-LIKE*; ***LlNRT1/PTR FAMILY 8.1-like*** *– NITRATE TRANSPORTER 1/PEPTIDE TRANSPORTER FAMILY 8.1-LIKE*; ***LlADO3*** *– ADAGIO PROTEIN 3*, ***LlALMT9-like*** *– ALUMINUM-ACTIVATED MALATE TRANSPORTER 9-LIKE*; ***LlMTERF2*** *– TRANSCRIPTION TERMINATION FACTOR MTERF2.*

		LlHSP2-like		***LlNRT1/PTR FAMILY 8.1-like***		***LlADO3***		***LlALMT9-like***		***LlMTERF2***	
**GENE SPECIFIC PRIMERS**		FP: CAATAATTTGTTAGGGAAATTTGAGC		FP: GGCAGTGCTGAGTGTTCTGA		FP: GTTCCGCCTCCTAGACTTGA		FP: TCCATTGACATGCCTGAATC		FP: GCTGCTAACATGAAAAAGTTGC	
		RP: CAGACAGTGATTTGAGGGACAC		RP: GCGGAGTGTTCCAATAGCTT		RP: TGGTGAGTGAAGACCTGCAA		RP: TCCATCACACAAACAGCACTC		RP: TGACAGTTTTGGCGCGTA	
**FLUORESCENCE DETECTION SYSTEM**		Hydrolysis probe		Hydrolysis probe		Hydrolysis probe		Hydrolysis probe		Hydrolysis probe	
**UPL PROBE NO**		125		49		119		69		39	
**qPCR PROGRAM:**											
**PRE-INCUBATION** x 1 cycle	Ramp rate 20 °C/s	95 °C	600 s	95 °C	600 s	95 °C	600 s	95 °C	600 s	95 °C	600 s
**AMPLIFICATION** x 45 cycles	DENATURATION	95 °C	10 s	95 °C	10 s	95 °C	10 s	95 °C	10 s	95 °C	10 s
	ANNEALING OF PRIMERS	52 °C	15 s	52 °C	15 s	52 °C	15 s	50 °C	15 s	48 °C	15 s
	EXTENSION	72 °C	1 s	72 °C	1 s	72 °C	1 s	72 °C	1 s	72 °C	1 s
**COOLING** x 1 cycle	20 °C/s	40°C	30 s	40°C	30 s	40 °C	30 s	40 °C	30 s	40 °C	30 s
